# Ascorbic acid and prunasin, two candidate biomarkers for endodormancy release in almond flower buds identified by a nontargeted metabolomic study

**DOI:** 10.1038/s41438-020-00427-5

**Published:** 2020-12-01

**Authors:** Jesús Guillamón Guillamón, Ángela Sánchez Prudencio, José Enrique Yuste, Federico Dicenta, Raquel Sánchez-Pérez

**Affiliations:** 1 Department of Plant Breeding. CEBAS-CSIC, Campus Universitario de Espinardo, 30100 Espinardo, Spain; 2 Metabolomics Platform of CEBAS-CSIC, Campus Universitario de Espinardo, 30100 Espinardo, Spain

**Keywords:** Plant breeding, Secondary metabolism

## Abstract

Temperate fruit trees belonging to *Prunus* species have the ability to suspend (induce dormancy) and resume growth periodically in response to environmental and seasonal conditions. Endodormancy release requires the long-term accumulation of chill. Upon accumulation of cultivar-specific chill requirements, plants enter the state of ecodormancy, which means the ability to grow has been restored, depending on the fulfilment of heat requirements. As many different metabolic pathways are implicated in endodormancy release, we have performed a metabolomic analysis, using the ultra-high-performance liquid chromatography–quadrupole time-of-flying (UPLC–QToF) technique. We assayed flower buds in different stages of endodormancy in four almond cultivars with different flowering times: the extra-early Desmayo Largueta, the late Antoñeta, the extra-late Penta, and the ultra-late Tardona. An orthogonal projection to latent-structure discriminant-analysis model was created to observe differences between endodormant and ecodormant flower buds. The metabolites showing the most significant variation were searched against the Metlin, HMDB, and KEGG libraries, which allowed us to identify 87 metabolites. These metabolites were subsequently assigned to specific pathways, such as abscisic acid biosynthesis, phenylpropanoid biosynthesis, and D-sorbitol metabolism, among others. The two metabolites that exhibited the most significant variations in all the cultivars studied with fold changes of up to 6.49 were ascorbic acid and prunasin. For the first time, these two metabolites have been proposed as potential biomarkers for endodormancy release in almond. Given the high synteny present between the *Rosaceae* species, these results could be extrapolated to other important crops like peach, plum, cherry, or apricot, among others.

## Introduction

Dormancy in temperate fruit species, like almond (*Prunus dulcis* (Mill.) D. A. Webb), is a defense state that allows trees to survive adverse conditions during winter. Endodormant buds are resistant to low temperatures^1^, whereas ecodormant buds, flowers, and young fruits are susceptible to frosts, which can result in important economic loss for farmers^2^. Dormancy is divided into three different stages: endodormancy, ecodormancy, and paradormancy^3^. Endodormancy, or winter dormancy, is controlled by the flower bud itself and is released by the accumulation of a certain quantity of chill. These requirements are specific for each cultivar and unless they are fulfilled, even under suitable climate conditions, the buds remain endodormant and unable to flower^4,5^. When endodormancy is released, growth is resumed upon response to warm temperatures during spring, a state defined as ecodormancy, which ends with flowering. In summer, buds enter paradormancy and stop their growth; this process is mainly governed by apical dominance. Finally, the dormancy cycle is closed when buds shift gradually to the endodormant stage in autumn^6^.

Given the importance of temperatures in the dormancy cycle, climate change can negatively affect the vegetative development, reproduction, and productivity of almond and other temperate tree species due to (1) global warming, which prevents chilling accumulation during winter^7,8^ and (2) late or spring frosts, which severely damage reproductive organs like ecodormant buds, flowers, and young fruits. During ecodormancy, the cold hardiness of *Prunus* species like sweet cherry (*Prunus avium* L.) and several other species is lower than that exhibited by other tree species^9^. Moreover, the frost resistance of peach (*Prunus persica* (L.) Batsch) and other *Prunus* species flower buds was found to decrease rapidly in response to heat accumulation during ecodormancy and even faster after a mild winter^10^.

Therefore, in order to counteract the effects of climate change in temperate fruit trees, breeding programs are focused on obtaining extra-late flowering cultivars with higher chilling requirements (CR) to prevent production loss due to late or spring frosts^11^. These chilling requirements are measured in mild-winter areas using the dynamic model (Chill Portions (CP))^4^. However, the breeding process is time- and cost-consuming, as it takes ~10–12 years to obtain a new almond cultivar. Moreover, climate change also slows down the fulfillment of CR of these cultivars, requiring the application of endodormancy-release agrochemicals to reach endodormancy release^12^. In order to assist breeding programs, and to better understand the molecular mechanisms underlying endodormancy release, several molecular studies have been performed. For many years, highly dense linkage maps have made it possible to identify quantitative trait loci (QTL) for flowering date and CR in almond and sweet cherry^13,14^. Later, genetic and transcriptomic studies have unveiled differentially expressed genes during endodormancy release, such as *DAM* (*DORMANCY-ASSOCIATED MADS-BOX*) family genes, which were described for the first time in peach^15^.

Many different metabolites that play a role in endodormancy maintenance and release have been described. Genes responsible for the synthesis of abscisic acid (ABA), for instance, are known to suffer a drop in expression, suggesting that ABA plays a crucial role as an endodormancy maintainer^16,17^. Other metabolites like sugars, implicated in the energy supply and developmental signals^18,19^, have also been suggested to play a role in endodormancy release. Moreover, further secondary or specialized metabolites, such as flavonoids, have been described as a vital factor related to endodormancy release in tomato, controlled by the gene *SlAN11*^20^. Redox metabolism also has a great impact on endodormancy release, since H_2_O_2_ decreases naturally in almond, Japanese pear (*Pyrus pyrifolia* (Burm. Fil.) Nakai), and other species after endodormancy release^21,22^. Finally, some Phe-derived metabolites like the cyanogenic glucoside prunasin and its derivatives might play an important function in endodormancy release in almond and sweet cherry, in which they show a significant increase during endodormancy release^23^.

Owing to the huge number of metabolites that could be involved in endodormancy release^24,25^, we have performed, for the first time, a nontargeted analysis during endodormancy in four almond cultivars whose flowering times range from extra-early to ultra-late and whose CR ranges from low (21 CP) to high (56 CP)^26^. As a result, we have been able to identify specific metabolite variations in one or more cultivars. We envisage that the variation in metabolites from different pathways found in this kind of study will provide a first step in finding a future biomarker for endodormancy release in almond and also in developing a modulator of endodormancy release, which will likely be applicable to other temperate fruit tree species, as well.

## Results

### Determination of endodormancy release

**Fig. 1:**
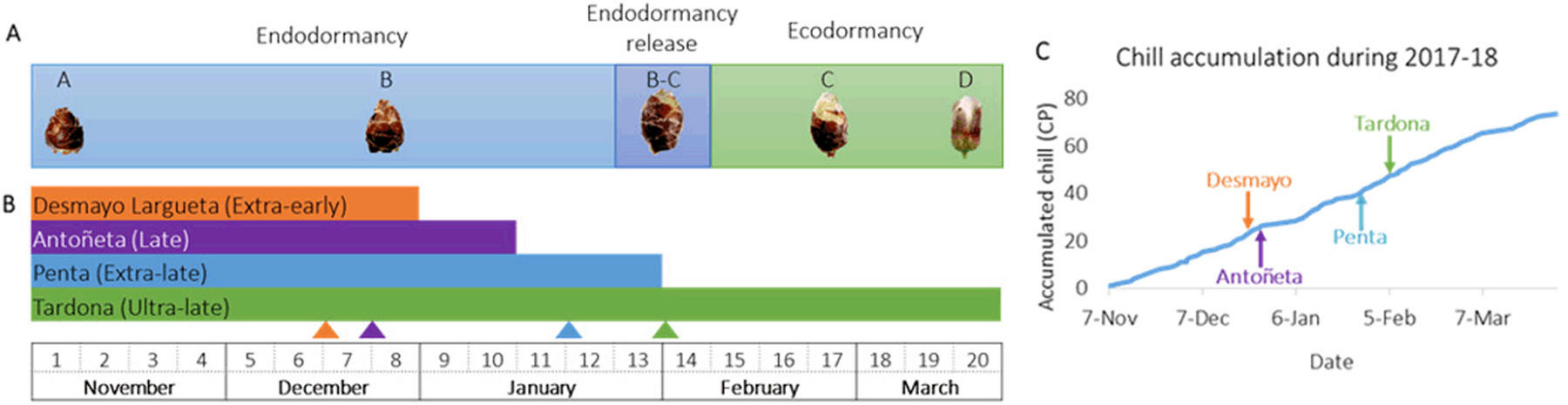
Sample-assay collection. **A** Phenological stages of Tardona in the different periods of dormancy, according to Felipe 1977^49^. **B** Diagram of the four studied cultivars from November 7 to March 21: orange for the extra-early Desmayo Largueta (D) cultivar, purple for the late Antoñeta (A) cultivar, blue for the extra-late Penta (P) cultivar, and green for the ultra-late Tardona (T) cultivar. **C** Chill accumulation during the endodormancy and ecodormancy period with the endodormancy-release date. For each cultivar, the dormancy-release time is marked with a triangle

Bud development in the growth chamber showed that the endodormancy-release date was December 19 for Desmayo Largueta, December 26 for Antoñeta, January 23 for Penta, and February 6 season 2017–2018 for the ultra-late Tardona cultivar (Fig. 1).

### Metabolomic profiling

In total, a set of 51 samples was analyzed for exhaustive cultivar fingerprinting. The model built is shown in Supplementary Fig. S1, in which we can observe the separation between endodormant and ecodormant buds on the x axis. Good-quality parameters for the model (Q^2^ > 0.84) were observed. The features that most contributed to the creation of the OPLS-DA were represented with a feature-importance plot (Supplementary Fig. S2). The model was validated using the OPLS-DA Overview (Supplementary Fig. S3). The separation between endodormant and ecodormant buds was mainly attributed to the fluctuation of 87 metabolites, which were remarkably significant according to a Volcano Plot analysis; of these 87 metabolites, 34 were detected in the negative-ionization mode and 53 in the positive-ionization mode (Supplementary Tables S3 and S4).

### Glutathione metabolism

**Fig. 2:**
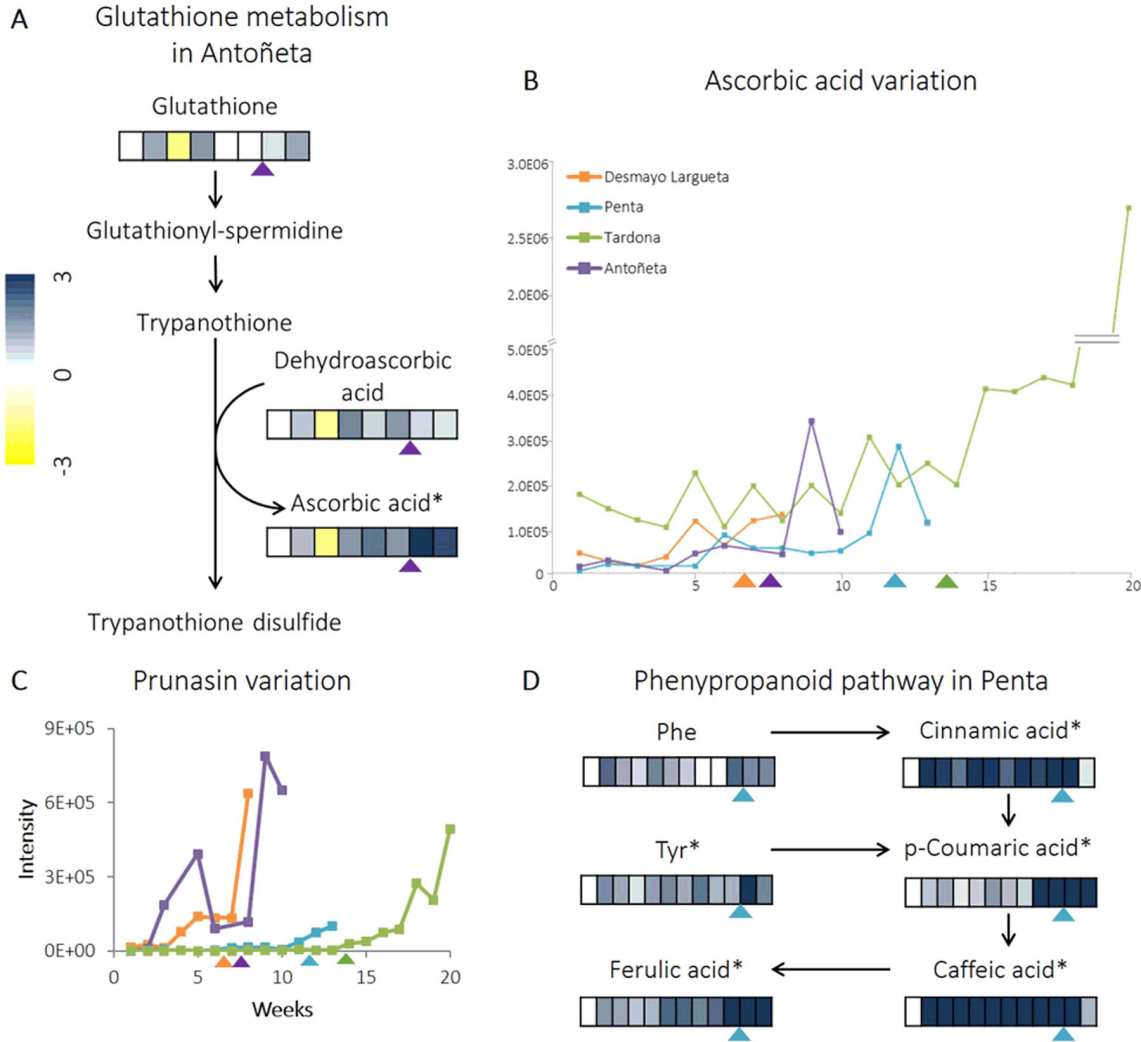
Glutathione metabolism and amino-acid-based pathways. **A** A heatmap showing the evolution of glutathione, dehydroascorbic acid, and ascorbic acid across the 8 weeks from endo- to ecodormancy evaluated in flower buds of the late cultivar Antoñeta. Values represent the means of FC from three biological replicates. **B** Ascorbic acid content variation in the four cultivar studied. A dashed line marks the dormancy-release date. Asterisks show the metabolites whose variation meets the requirements for being considered significant. **C** Variation of prunasin content in the four cultivar studied. **D** Phenylpropanoid-pathway heatmap showing the evolution of cinnamic acid, p-coumaric acid, caffeic acid, ferulic acid, and their precursors Phe or Tyr, across the 12 weeks from endo- to ecodormancy in the flower buds of the extra-late Penta cultivar. Values represent the means of FC from three biological replicates. Triangles mark the dormancy-release date. Asterisks show the metabolites whose variation meets the requirements for being considered significant. The extra-early Desmayo Largueta is in orange, the late Antoñeta in purple, the extra-late Penta in blue and the ultra-late Tardona in green

Ascorbic acid, a by-product in glutathione metabolism, showed a sharp increase (FC > 2) during endodormancy release in the extra-early Desmayo Largueta, the extra-late Penta, and the ultra-late Tardona, while it varied right after endodormancy release in the late Antoñeta (Fig. 2A, B). Additionally, we managed to detect a small increase in dehydroascorbic acid and glutathione (GSH), but it was not as significant as that seen in ascorbic acid.

### Amino-acid-based pathways

The first specialized metabolite was prunasin, a Phe-derived metabolite (Fig. 2C), which showed a strong increase (FC > 3) during and after the endodormancy release in the four cultivars analyzed.

Other Phe-derived metabolites identified to belong to the phenylpropanoid biosynthetic pathway, where most of the metabolites and intermediates presented a significant increase. This could be seen in the extra-late Penta cultivar, whose endodormancy-release date was estimated in week 12 of sampling, whereas the other cultivars did not present such variations (Fig. 2D). p-Coumaric acid showed an FC of 5.48 between weeks 9 and 10, two weeks before endodormancy was released.

Similarly, ferulic acid showed a steady increase and reached a maximum during weeks 11 and 12 with an FC of 4.51. In contrast, from the beginning of endodormancy, caffeic aid and cinnamic acid exhibited a similar increase, with an FC of 3.46 and 3.03, respectively. Therefore, cinnamic acid, p-coumaric acid, caffeic acid, and ferulic acid all peaked at the moment of endodormancy release, followed by a sharp drop, with the exception of p-coumaric acid, which kept increasing after endodormancy release (Fig. 2D). It is remarkable that almost all of the compounds experienced a great increase over time, even presenting FCs over four. In contrast, no significant variation was observed in their precursors, Tyr and Phe.

The biosynthesis of Pro showed a significant fluctuation in the ultra-late Tardona cultivar, while it remained invariable in the other cultivars (Supplementary Fig. S4A). Glu also fluctuated, but in the opposite way, suffering drops, while Pro experienced an increase. Neither fluctuation was relevant, however. On the other hand, the intermediate metabolite 1-pyrroline increased during endodormancy release with an FC of 2.08.

The last amino acid detected was Trp, which presented some variation in the extra-early Desmayo Largueta cultivar (Supplementary Fig. S4B). The amount of Trp fluctuated during endodormancy, increasing with the release of endodormancy to a maximum FC of 1.94. On the contrary, the Trp-derived metabolites like tryptamine showed a gradual decrease over time, down to an FC of −4.14.

A similar pattern to that observed for prunasin could be observed in 2-methylbutylamine (Supplementary Fig. 4C), which is a by-product of Ile degradation. This compound showed a huge increase just before endodormancy release in the extra-early Desmayo Largueta and the extra-late Penta cultivars only.

### The ABA biosynthetic pathway

**Fig. 3:**
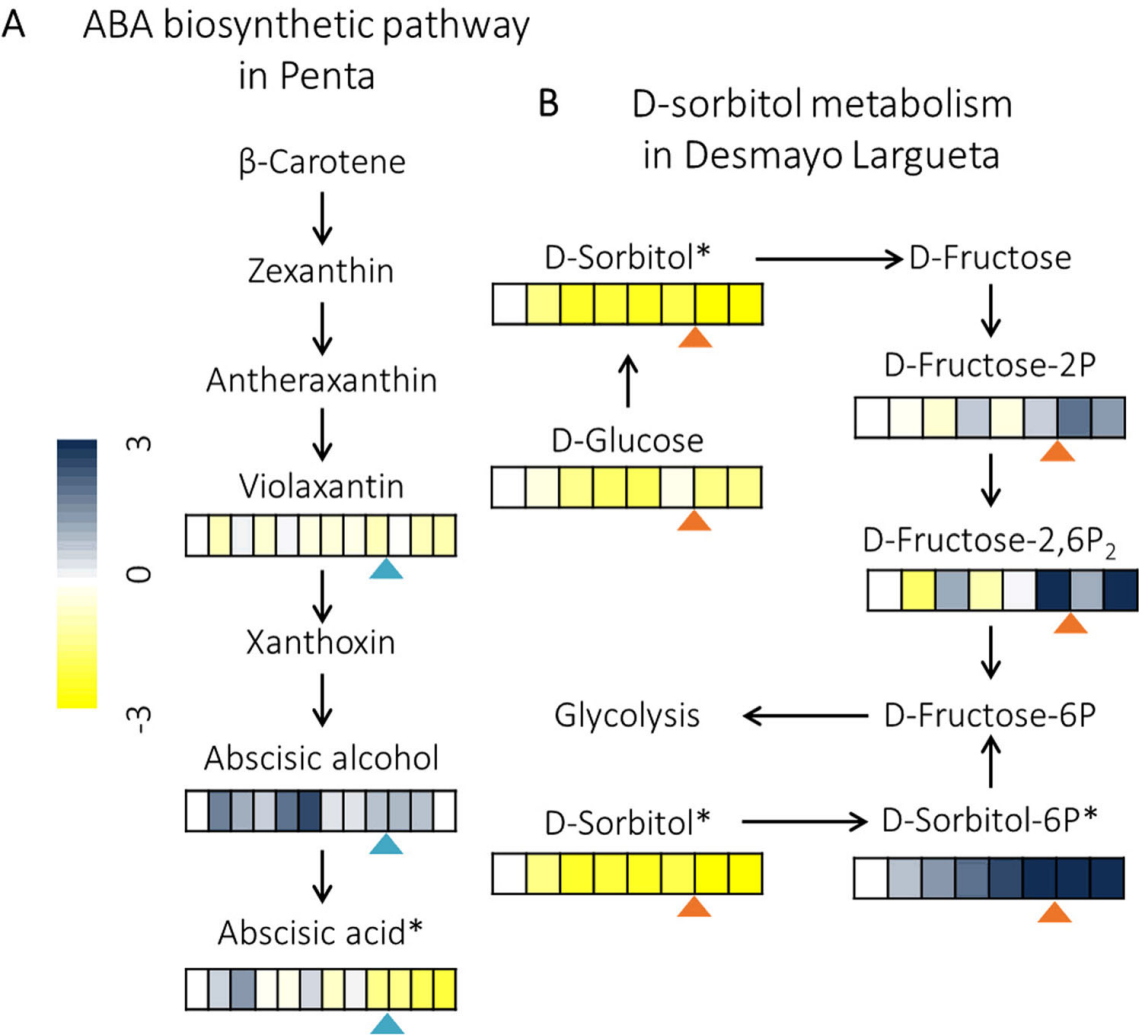
Abscisic acid (ABA) biosynthetic pathway and D-sorbitol metabolism. **A** A heatmap showing the variation of violaxanthin, abscisic alcohol, and ABA across the 12 time points from endo- to ecodormancy in the flower buds of the extra-late Penta cultivar. Values represent the means of FC from three biological replicates. A dashed line marks the dormancy-release date. Asterisks show the metabolites whose variation meets the requirements for being considered significant. **B** A heatmap showing the variation of D-glucose, D-sorbitol, D-fructose-2-P, D-fructose-2,6-P_2_, and D-sorbitol-6-P across the eight time points from endo- to ecodormancy in the flower buds of the extra-early Desmayo Largueta cultivar. Values represent the means of FC from three biological replicates. Triangles mark the dormancy-release date. Asterisks show the metabolites whose variation meets the requirements for being considered significant

In the biosynthesis of ABA, metabolites like violaxanthin, abscisic alcohol, and ABA presented variations in the extra-late Penta cultivar (Fig. 3A), but no variations in the rest of the cultivars. ABA showed a gradual and significant drop at the end of endodormancy, falling to an FC of −2.11, whereas variations in violaxanthin and abscisic alcohol were not significant.

### D-sorbitol metabolism

In the sorbitol metabolism, variations were detected in five different metabolites in the extra-early Desmayo Largueta cultivar (Fig. 3B) that remained constant in the other cultivars. Sorbitol and sorbitol-6-phosphate, with FCs of −4.18 and 2.84, respectively, experienced a huge drop and increase. D-glucose also exhibited a dramatic drop upon endodormancy release. Consequently, D-sorbitol-6-phosphate experienced a huge rise, as did the immediate precursors of the glycolysis, D-fructose-2,6-biphosphate and D-fructose-2-phosphate, which showed an FC of 2.06 and 1.23, respectively.

### Flavonoids

Two different groups of flavonoids were identified among almond samples. The first was anthocyanins, in which case two different metabolites were found. The anthocyanin petunidin-3-glucoside presented an FC > 3 in the extra-early and in the extra-late cultivars. We could also detect malvidin-3-glucoside, although its variation was not significant (Supplementary Fig. S5A and B). The second group was flavonols, in which case flavonol quercetin-3-(6”-malonylglucoside) fluctuated in the extra-early Desmayo Largueta cultivar only with an FC of 2.10 (Supplementary Fig. S5C).

### Metabolite–metabolite correlation analysis during endodormancy release

**Fig. 4:**
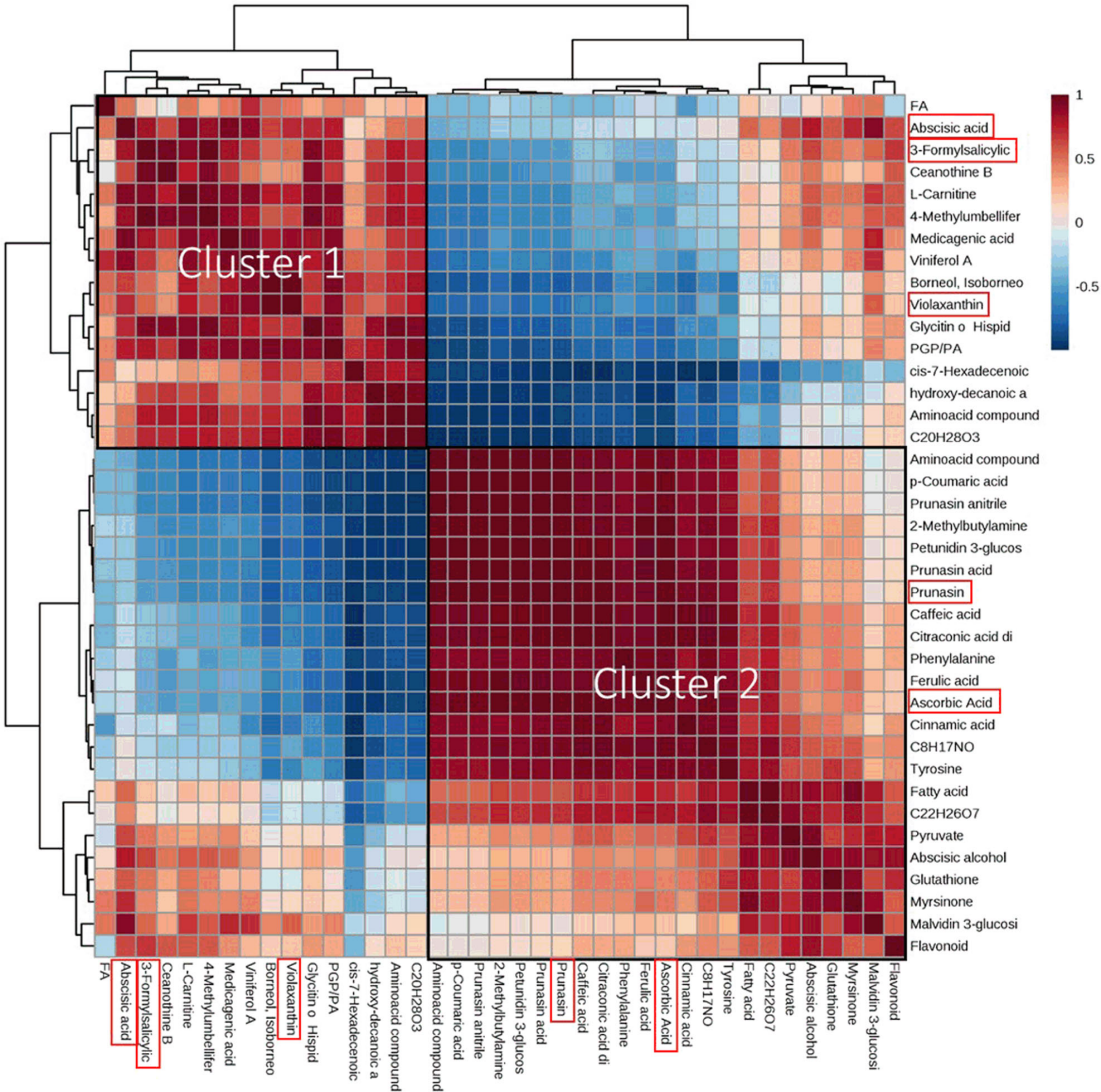
Correlation heatmap cluster of the extra-late Penta cultivar. Two different clusters of metabolites are shown, grouping together either the metabolites that are in the same pathway or the metabolites that present the same kind of variation. The complete name of the metabolites with ~ can be found in Supplementary Table S3

The correlation heatmap of the extra-late cultivar Penta showed two different clusters (Fig. 4). Cluster 1 grouped together metabolites belonging to the same pathway, such as violaxanthin and ABA, with a deep correlation with 3-formylsalicylic acid; cluster 2 grouped together metabolites showing the same trend, like those involved in the phenylpropanoid biosynthesis pathway, ABA biosynthesis, glutathione metabolism, or anthocyanin biosynthesis (cluster 2).

We also observed two different clusters in the correlation heatmaps of the extra-early cultivar Desmayo Largueta (Supplementary Fig. S6) and the late cultivar Antoñeta (Supplementary Fig. S7); the ultra-late Tardona cultivar, on the other hand, showed just one cluster (Supplementary Fig. S8). As in the heatmap of the extra-late Penta cultivar, we could observe that metabolites from the same pathways and with similar variation patterns were located in the same cluster.

## Discussion

### Glutathione metabolism

Previous studies have explained the importance of glutathione metabolism and especially ascorbic acid as an antioxidant metabolite against ROS during endodormancy. For example, in Japanese pear and sweet cherry, ROS increases during endodormancy and decreases afterward upon endodormancy release^25,27^. In our study, a significant increase in ascorbic acid was observed in the four cultivars studied (Fig. 2B). Future studies should focus on ROS measurement to confirm whether this also happens in almond. Moreover, our results are in concordance with the previous findings in Japanese pear and other species, where the activity of ascorbate peroxidase increased during endodormancy release. This fact indicates that the transition of the endodormancy stage may involve the removal of free radicals through a system using ascorbic acid as an antioxidant by ascorbate peroxidase^27^. It has been demonstrated in grapevine leaves (*Vitis vinifera* L.) that a rise of the ascorbic acid-level group with an increase in flavonols is implicated in the degradation of H_2_O_2_^28^. This agrees with the trend shown by these metabolites in our samples, where their levels increased during endodormancy release in order to decrease ROS.

Furthermore, ascorbic acid has also been described as being related to the ABA-gibberellin (GA) balance, implicated in seed germination in rice (*Oryza sativa* L.). In fact, an ABA increase with a concomitant decrease in GA produced a drop in ascorbic acid, keeping the seed dormant^29^. On the contrary, we could observe an increase in ascorbic acid in all the cultivars assayed; moreover, this increase was accompanied by a simultaneous drop in ABA in the extra-late Penta cultivar. The relationship described between ascorbic acid and ABA in rice seeds agrees with our observations, since, in seeds, an ABA increase is responsible for keeping the seed dormant, but in flower buds, ABA levels must decrease in order to reach endodormancy release. This hypothesis will be validated in further studies with more cultivars. Overall, ascorbic acid represents a potential biomarker for endodormancy release in almond.

### Amino-acid-based pathways

As previous studies in almond and sweet cherry^23^ have shown, prunasin increases during and after endodormancy release (Fig. 2C). Furthermore, those studies found a concomitant increase in prunasin anitrile and prunasin amide^23^, which we did not find in our study. All things considered, prunasin could be used as a potential biomarker for endodormancy release.

In peach, it has been described that the accumulation of phenylpropanoids coupled with a drop in ABA is necessary for reaching endodormancy release^30^, as shown in the extra-late Penta cultivar (Fig. 2D). Moreover, in sweet cherry flower buds, caffeic acid has been found to increase in the early stages of endodormancy and drop right after endodormancy release^31^, in agreement with our observations in the extra-late Penta cultivar (Fig. 2D). However, these authors did not find a cinnamic acid increase as we did. In contrast, other metabolites like p-coumaric acid and ferulic acid increased before, during, and 2 weeks after endodormancy release, indicating a potential role as biomarkers for these specialized metabolites in the transition from endodormancy to ecodormancy. In fact, the *4-COUMARATE:CoA LIGASE-LIKE 1* (*4-CLL1*) gene has recently been described in almond endodormancy release (Prudencio et al. 2020^32^). This tendency indicates that a large concentration of this metabolite is still necessary after endodormancy release, suggesting its metabolization into other products such as its CoA derivatives. Very recently, a homolog to this gene has been described in apricot (*Prunus armeniaca* L.), the *4-CLL7* gene, which shows a steep increase during and after endodormancy release^33^.

Previous studies in grapevine have pointed to Pro as an osmotic factor that could protect flower buds against dehydration during winter^34^. This could explain the accumulation of 1-pyrroline, the immediate precursor of Pro, observed in the ultra-late Tardona cultivar (Supplementary Fig. S4A).

Finally, the Trp increase during endodormancy release (Supplementary Fig. S4B) is in concordance with previous findings in sweet cherry flower buds^31^. This increase could be caused by the high level of the activity-dormancy cycle, during which some compounds that varied during endodormancy are metabolized into others involved in growth and other physiological processes, like indole-3-acetic acid (IAA), a Trp-derived specialized metabolite^35,36^. However, this disagrees with some previous findings in sweet cherry, where genes responsible for the biosynthesis of IAA, such as *YUC10*, were downregulated during endodormancy release^12^. In *Arabidopsis thaliana*, strictosidine synthase has also been found to play a crucial role in the microsporogenesis process, during which strictosidine is needed for pollen-grain development^10^. In our study, this metabolite did not increase, probably due to the fact that this study was focused on the dormancy period and not on flowering. It has also been described in different apricot cultivars that microsporogenesis is related to endodormancy released, suggesting that an early microsporogenesis might be involved in a low rate of blooming^37^.

2-Methylbutylamine, a by-product of Ile metabolization^38^, showed a steep increase in the extra-early Desmayo Largueta cultivar and a gradual increase in the extra-late Penta cultivar (Supplementary Fig. S4C). To the best of our knowledge, this is the first time that this metabolite has been described in the endodormancy-release process.

### The ABA biosynthetic pathway

ABA has been described as playing a crucial role in endodormancy release, and ABA levels have been found to decrease in order for plants to reach the ecodormancy phase^39^; these findings agree with our observations in the extra-late Penta cultivar (Fig. 3A). Additionally, ABA has also been described as being implicated in the modulation of other metabolic pathways that we have studied, such as glutathione metabolism, phenylpropanoid biosynthesis, and flavonoid biosynthesis^29,30,40^.

### D-sorbitol metabolism

In peach flower buds, the accumulation of sorbitol is activated by chill, inhibiting the expression of the sorbitol catabolic genes^41^. This agrees with our results in the three cultivars with the highest CR (Antoñeta, Penta, and Tardona) (Fig. 1C and Supplementary Table S1), in which D-sorbitol may be acting as an osmolite and protecting flower buds against dehydration during winter. Nevertheless, a huge decrease in D-sorbitol with the concomitant accumulation of D-sorbitol-6-P was observed in the extra-early Desmayo Largueta cultivar (Fig. 3B), whose CR for endodormancy release is low. Moreover, the glycolysis metabolites D-fructose-2-P and D-fructose-2,6-P_2_ increased in the stages close to endodormancy release (Fig. 3B). It is known that during endodormancy release in red-rice seeds, the glycolysis and energy obtained increase^42^; these findings agree with the increase in the compounds from D-sorbitol metabolism that we could observe in the extra-early Desmayo Largueta cultivar, suggesting the role this metabolite plays in early endodormancy release.

### Flavonoid metabolism

In sheepgrass (*Leymus chinensis* (Trin.) Tzvelev), during seed germination, high ABA levels downregulate anthocyanin biosynthesis through the inhibition of anthocyanidin synthase and anthocyanidin reductase by the *bHLH92* transcription factor^40^. In our study, we observed a drop in ABA with a concomitant increase in the anthocyanin petunidin-3-glucoside during endodormancy release (Supplementary Fig. S5A, B). We, therefore, suggest that the anthocyanin increase seen in our samples is probably involved with the ABA drop previously explained in the extra-late Penta cultivar (Fig. 3A). Regarding flavonol biosynthesis, it has been described that under low temperatures, some flavonol-associated genes are repressed in apple (*Malus domestica* (Suckow) Borkh.)^43^. Accordingly, we were able to detect that flavonol biosynthesis increased during endodormancy release (Supplementary Fig. S5C), since once CR has been fulfilled, the flower bud is ready to awake and the genes are no longer downregulated by the chill.

### Metabolite–metabolite correlation analysis during endodormancy release

**Fig. 5:**
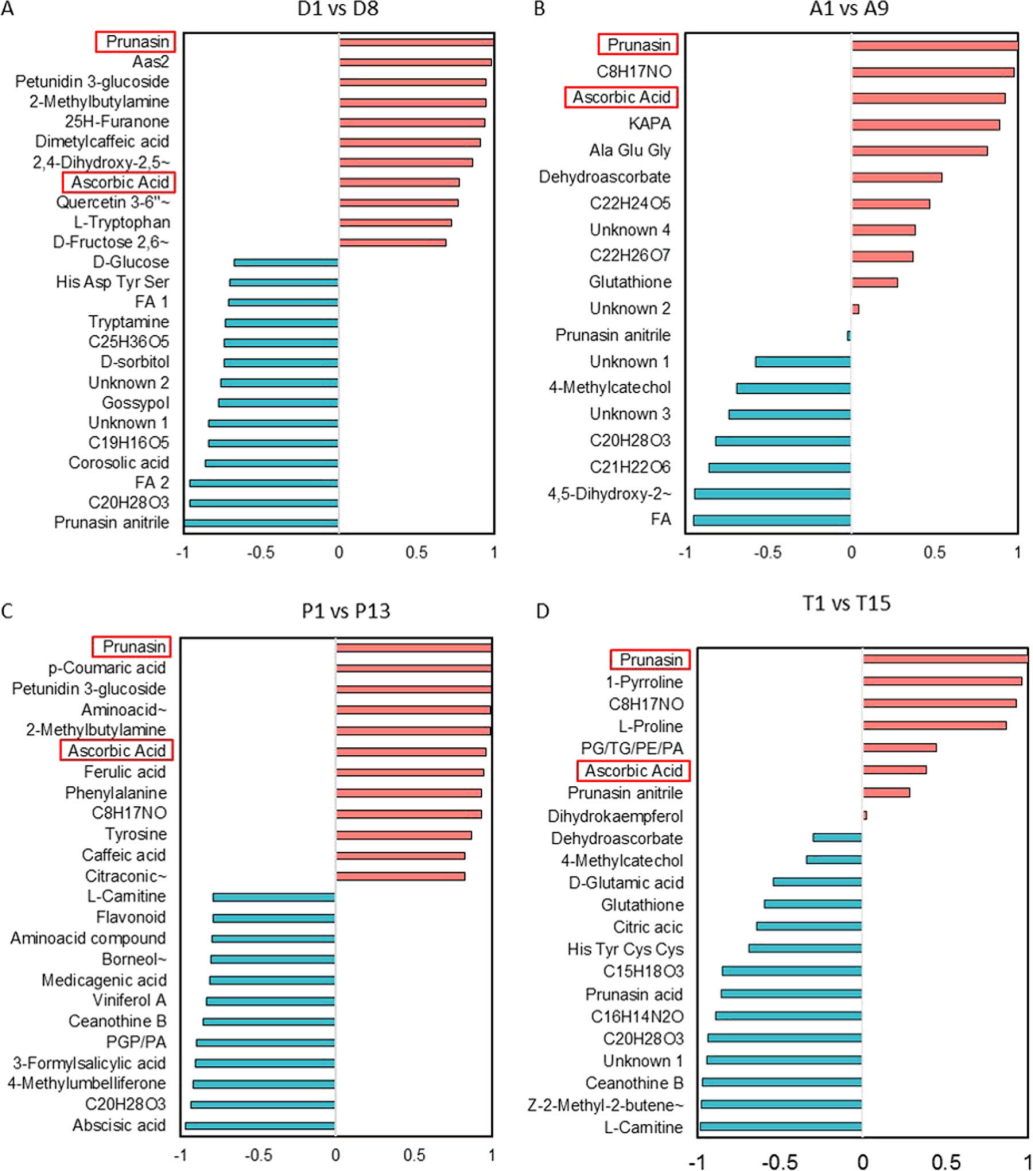
Pattern-searching plot between prunasin and the metabolites that showed variation from endo- to ecodormancy in the four cultivars studied. Up to 25 of the top metabolites were grouped together with positive (salmon) or negative (cyan) correlation in the extra-early Desmayo Largueta (**A**) comparing stage 1 and stage 8 (D1 vs D8), in the late Antoñeta (A1 vs A9) (**B**), in the extra-late Penta (P1 vs P13) (**C**), and in the ultra-late Tardona (T1 vs T15) (**D**)

Correlation heatmaps showed how metabolites varied with respect to one another (Fig. 4 and Supplementary Figs. S6–S8), showing that metabolites with similar trends during endodormancy released clustered together. In order to corroborate the correlation between prunasin and the rest of the metabolites previously identified in the four cultivars, a pattern-searching plot was created facing endodormant (D1, A1, P1, and T1) against ecodormant (D8, A9, P13, and T15) flower buds (Fig. 5). A great number of metabolites showed a huge correlation with prunasin in all of our cultivars. The high level of relationship between ascorbic acid and prunasin—the only two metabolites that varied significantly in all our cultivars—was especially remarkable. The increases we observed in ascorbic acid and prunasin present a similar variation pattern (Fig. 2A–C). In fact, in peach, it has been described that both metabolites are connected under salt-stress scenarios, during which increases in salicylic acid (SA) and ABA cause a drop in cyanogenic glucoside and ascorbic acid levels^44,45^. This connection might also exist in the almond endodormancy-release process: in the extra-late Penta cultivar, ABA and 3-formylsalicylic acid (SA precursor) levels decreased (Fig. 3A), whereas ascorbic acid and prunasin levels increased (Fig. 2A–C, respectively), with a high negative correlation between prunasin, ABA, and the SA precursor.

Besides, prunasin exhibited a high correlation with metabolites with similar behavior in the extra-early Desmayo Largueta cultivar (Fig. 5A), such as the flavonoids petunidin-3-glucoside, which also showed a high correlation in the extra-late Penta cultivar, and quercetin-3-(6 “-malonylglucoside); both flavonoids have been associated with seed germination and endodormancy release in sheepgrass and apple^40^. Trp and tryptamine also exhibited a high positive and negative correlation, respectively, with prunasin. The opposite correlation directions are probably due to the different trends of each metabolite (Supplementary Fig. S4), since tryptamine decreased during endodormancy release while Trp increased. This Trp behavior is probably due to the high level of the activity-dormancy cycle in this period^35,36^.

Prunasin acid, described for the first time in dormant and nondormant germinated seeds^46^, showed the highest negative correlation detected in the extra-early Desmayo Largueta cultivar (Fig. 5A). The same results were previously observed in the flower buds of the extra-early almond cultivars Achaak and Desmayo Largueta, while prunasin acid levels were minute in the extra-late cultivar as Penta^23^.

In the late Antoñeta cultivar (Fig. 5B), the unknown metabolite with the molecular formula C_8_H_17_NO, which is also found in the extra- and ultra-late cultivars Penta and Tardona, presented a high correlation with prunasin, suggesting that this metabolite might be associated with endodormancy release in late cultivars. Further studies should focus on the identification of this compound. KAPA, 7-keto-8-aminopelargonic acid, an Ala derivative in the biotin biosynthesis found in *A.* *thaliana*^47^, also exhibited a high correlation with prunasin. To the best of our knowledge, this is the first time that this metabolite has been described in the endodormancy-release process. With a high negative correlation, an unknown fatty acid (FA) was also found. FAs have been described as a source of ROS, either directly via β-oxidation or by an additional step of neoglucogenesis, producing monosaccharides^48^. In sweet cherry flower buds, hydrogen cyanamide treatment used to advance flower-bud dormancy decreased the expression of transcripts coding for the ROS-scavenging enzyme catalase 1 (CAT1) and manganese-containing superoxide dismutase (Mn-SOD)^12^. This agrees with the catalase-activity decrease observed in our study. Consequently, an increase in ROS, like H_2_O_2_, was found to be related to endodormancy release in sweet cherry^12^.

The highest correlations between prunasin and other metabolites were observed in the extra-late Penta cultivar (Fig. 5C); primary metabolites like Phe and Tyr and their derivatives, the phenylpropanoids p-coumaric acid, ferulic acid, and caffeic acid, were among the metabolites that presented the highest correlation with prunasin. The phenylpropanoid pathway has recently been described as varying during endodormancy release in five apricot cultivars, in which genes responsible for the biosynthesis of cinnamoyl CoA and p-coumaroyl-CoA exhibited a FC higher than 3.5^33^. This trend is similar to that observed for cinnamic acid and p-coumaric acid in our samples, as well as to that found in prunasin; this fact would explain the high correlation between these metabolites and prunasin.

Finally, in the ultra-late Tardona cultivar (Fig. 5D), 1-pyrroline and Pro presented a high correlation with prunasin. Pro has been defined as being an osmotic protector during endodormancy release in grapevine flower buds^34^. With a negative correlation, compounds like prunasin acid and L-carnitine were detected. Prunasin acid results are in concordance with observations in previous studies in flower buds of the late S3067 and the extra-late Penta cultivars^23^. L-carnitine correlation was not only found in the ultra-late Tardona but also in the extra-late Penta, suggesting a possible link with the delayed flowering time. In peach, proteins responsible for carnitine transport were enhanced in endodormant flower buds^10^.

## Conclusion

**Fig. 6:**
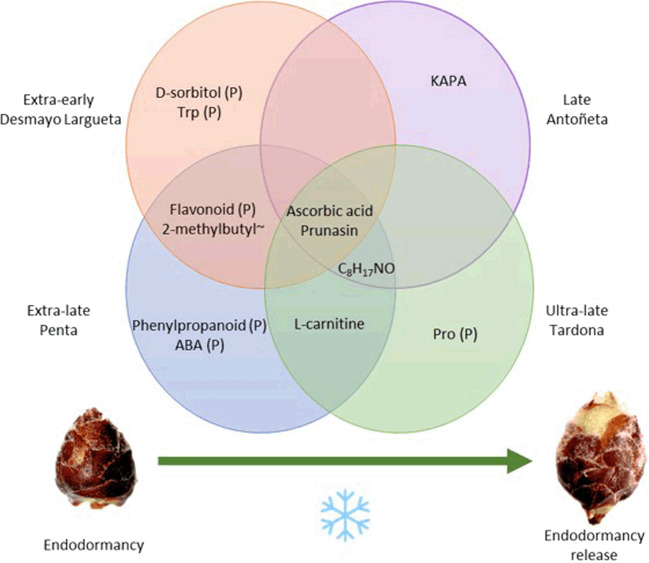
Pathways and metabolites involved in endodormancy release. Venn diagram shows the metabolites with a significant variation in one or more cultivars and the pathways (P) in which a metabolite varied significantly. The complete names of metabolites with ~ can be found in Supplementary Table S3

Production in temperate fruit species like almond relies on successful flowering, which only occurs upon adequate endodormancy release. Monitoring this multifaceted process is therefore crucial to assure high productivity. With this nontargeted metabolomic study, 87 compounds were identified; out of these compounds, prunasin and ascorbic acid were found to be significantly present in the four almond cultivars studied. Based on these results, the two best candidate biomarkers for endodormancy release in almond are prunasin and ascorbic acid. Further studies should focus on validation using more cultivars with different endodormancy-release requirements and in other almond-related species like apricot, peach, or sweet cherry, among others, but also considering different-year observation. The discovery of a biomarker for endodormancy release and the metabolic process behind it (Fig. 6) would suppose a great advance in monitoring the process, making it possible to predict the endodormancy-release date. Under unfavorable climate conditions, the biomarkers could boost the development and application of environmentally friendly modulators for endodormancy release, bringing forward or delaying the flowering time. This would counteract the effects of climate change, improving efficient and sustainable fruit production.

## Materials and methods

### Plant material

For this study, we used the following four almond cultivars from the experimental field of CEBAS-CSIC (Murcia, Spain; latitude: 38° 5’ 54.38” N, longitude: 1° 2’ 3.18” W): Desmayo Largueta (21- year-old tree grafted onto Garrigues seedling rootstock), Antoñeta (20-year-old tree grafted onto Garrigues seedling rootstock), and Penta and Tardona (17-year-old tree grafted onto Garrigues seedling rootstock). Desmayo Largueta is a traditional Spanish extra-early flowering cultivar, whereas Antoñeta (Ferragnès × Tuono), Penta (S5133 × Lauranne), and Tardona (S5133 × R1000) are late, extra-late, and ultra-late cultivars^11^, respectively, from the CEBAS-CSIC almond- breeding program^11,26^ (Supplementary Table S1). For each cultivar assayed, flower buds were collected weekly in three independent biological replicates from the same tree. Sampling started on Nov 7 (stage A = all cultivars fully endodormant) and lasted until March 21 (stage D = ecodormant stage in Tardona)^49^, covering the endodormant-to-ecodormant stage for each of the cultivars. In the case of Tardona, the sampling period was extended to stage D, previous to flowering time, to study the evolution of the metabolites that varied significantly during endodormancy and ecodormancy. In total, we collected 51 samples, which were labeled as D1–D8 for Desmayo Largueta, A1–A10 for Antoñeta, P1–P13 for Penta, and T1–T20 for Tardona (Fig. 1). Each cultivar has a different number of samples due to the different time periods required to reach endodormancy release.

### Determination of endodormancy release

The phenological stages of the flower buds were determined weekly using the forcing method as described by Prudencio et al.^26^ (Fig. 1). Three branches consisted of single mixed twigs; 1-year shoots with flower and vegetative buds (40-cm long and 5 mm in diameter) of each cultivar were picked and placed in a growth chamber (23 ± 1 °C, RH 40% during the 16 h of photoperiod and 20 ± 1 °C, RH 50% during the dark period). After making a fresh cut at the base of each branch, the branches were placed in a 5% sucrose solution with 0.1% aluminum sulfate in the growth chamber. After 10 days in the growth chamber, the phenological state of flower buds on the branches was evaluated. When 50% of the flower buds were in the B–C state^49^, the date of endodormancy release was established as it has been previously described^13,50^. Additionally, the accumulated chill during winter was measured using the dynamic model suitable for mild-winter areas^4^ (Fig. 1C and Supplementary Table S1).

### UPLC–QToF–MS/MS analysis

All flower-bud samples collected (except P4 and A7) (three 50–100-mg replicates) were ground to a fine powder in liquid nitrogen, mixed with 200 µl of ACN:H_2_O (80:20) with 0.1% HCOOH (*Sigma*), mechanically shaken, sonicated for 3 × 30 s, and centrifuged for 10 min at 13,000×*g*, as previously described^51^. The supernatant was stored at −20 °C until further analysis. Samples were mixed with glipizide (*Sigma*) at 0.1 µg/ml as an internal standard and passed through a filter plate (0.22 µm, *Millipore*) by centrifugation (5 min, 1107×*g*).

UPLC–QToF–MS/MS was performed using a Waters ACQUITY UPLC I-Class System (*Waters Corporation*, Milford, MA, USA) coupled to a Bruker Daltonics QToF-MS mass spectrometer [*maXis impact Series* with a resolution ≥ 55000 FWHM *Bruker Daltonics*, Bremen, Germany], using ESI for both positive- [ESI ( + )] and negative- [ESI (−)] ionization modes.

UPLC separation was performed using an HSS T3 C18 100 × 2.1-mm column with 1.8 µm of size particle (*Waters Corporation*, Milford, MA, USA) at a flow rate of 0.3 mL/min. The separation was performed using H_2_O with formic acid at 0.01% (pH ~3.20) (*PanReac AppliChem*, Barcelona, Spain) as the weak mobile phase (A) and ACN with HCOOH at 0.01% (J. T. Baker, New Jersey, USA) as the strong mobile phase (B). The gradient started with 10% of B at 0 min, which was progressively increased up to 90% at minute 14; after that, it was held until minute 16 and then decreased to 10% at 10 s, where it remained until minute 18 (Supplementary Table S2). Nitrogen was used as the desolvation gas with a flux of 8 L/min and as the nebulizing gas with a pressure of 2.0 bar. The drying temperature was 200 °C and the column temperature was 40 °C. The voltage source was 4.0 kV for ESI (−) and 4.5 kV for ESI ( + ). The MS experiment was carried out using HR-QTOF-MS, applying 24 eV for ESI ( + ) and 20 eV for ESI (−) and using broadband collision-induced dissociation (bbCID). MS data were acquired over an *m/z* range of 45–1200 Da.

The external calibrant solution was delivered by a KNAUER Smartline Pump 100 with a pressure sensor (*KNAUER*, Berlin, Germany). The instrument was calibrated externally before each sequence with a 10 mM sodium formate solution. The mixture was prepared by adding 0.5 mL of formic acid and 1.0 mL of 1.0 M sodium hydroxide to an isopropanol/Milli Q water solution (1:1, v/v).

### Data processing and statistical analysis

We were able to start obtaining the raw-intensity data of the analysis from the nontarget metabolomics data workflow using Profile Analysis 2.1 software (*Bruker Daltonics*); we obtained 654 features with a signal-to-noise ratio of 3 for ESI (−) and 6218 features with a signal-to-noise ratio of 5 for ESI ( + ). For statistical analysis, we used *MetaboAnalyst 4.0* software (http://www.metaboanalyst.ca/MetaboAnalyst/faces/home.xhtml). We transformed our data in a logarithmic base and performed a Pareto scaling. Likewise, we made an Orthogonal Partial Least‐Square Discriminant Analysis model (OPLS-DA) (Supplementary Fig. S1), which is a supervised model, in order to test whether there were real differences between endodormant and ecodormant flower buds. Furthermore, to test the robustness of the model, we used the OPLS-DA overview to obtain the explained variance and the capability of prediction, represented by the *R*^2^ and *Q*^2^ scores, respectively (Supplementary Fig. S2). In addition, we created a feature-importance plot based on the covariance and the correlation of the features of the model. Finally, with the aim of observing which features presented significant variation between endodormant and ecodormant flower buds, we performed a Volcano plot analysis based on the *P* value and the fold change of the features of our samples. The statistical significance was set to a logarithm of fold change (FC) > 2 and a *P* value <0.05.

### Metabolite identification

The most significant metabolite MS/MS spectra were acquired and searched in the following databases: METLIN (https://metlin.scripps.edu/landing_page.php?pgcontent=mainPage), the Human Metabolome Database (http://www.hmdb.ca/), and Lipid Maps (https://www.lipidmaps.org/). Additionally, we used prunasin, prunasin acid, prunasin anitrile, and amygdalin standards for the identification of those metabolites^23^. Subsequently, the KEGG PATHWAY database (https://www.genome.jp/kegg/pathway.html) was used to identify the different pathways the compounds belonged to. We also studied the behavior of the metabolites that were in the same pathway of metabolites showing significant variation, even if their variation was not significant.

Finally, to determine the statistical correlation between the significant compounds involved in the pathways, we measured the distance between our metabolites with a Pearson *r* correlation coefficient, creating a correlation-analysis heatmap and a pattern-searching plot using MetaboAnalyst 4.0 software.

## Supplementary information

Supplemental Material
