# Supplementary material for: Ascorbic acid and prunasin, two candidate biomarkers for endodormancy release in almond flower buds identified by a nontargeted metabolomic study
Source: Hortic Res. 2020 Dec 1;7:203. doi: 10.1038/s41438-020-00427-5 (PMC7705690; doi:10.1038/s41438-020-00427-5)
Supplement: Supplementary file 1 — Supplemental Material [file 41438_2020_427_MOESM1_ESM.docx]

**Supplemental table S1.** Almond cultivars assayed including the pedigree, origin and main agronomic characteristics.

| **Cultivar** | **Pedigree** | **Origin** | **Flowering** | **Flowering Date (Julian)** | **CR (Chill portions)** |
| --- | --- | --- | --- | --- | --- |
| Desmayo Largueta | Unknown | Spain, traditional cultivar | Extra-early | 29 | 20.4 |
| Antoñeta | Ferranès x Tuono | Spain, breeding release CEBAS-CSIC | Late | 55 | 25.3 |
| Penta | S5133 x Lauranne | Spain, breeding release CEBAS-CSIC | Extra-late | 76 | 37.5 |
| Tardona | S5133 x R1000 | Spain, breeding release CEBAS-CSIC | Ultra-late | 87 | 47.3 |

**Supplemental table S2.** Gradient applied for the LC-MS analysis.

| **Time (min)** | **Flux (mL/min)** | **% Mobile phase A** | **% Mobile phase B** |
| --- | --- | --- | --- |
| 0 | 0.3 | 90 | 10 |
| 14 | 0.3 | 10 | 90 |
| 16 | 0.3 | 10 | 90 |
| 16.1 | 0.3 | 90 | 10 |
| 18 | 0.3 | 90 | 10 |

**Supplemental table S3.** Tentative identification of the studied metabolites based on their m/z ratio, molecular formula, adduct and ionization mode, data base used and ΔPPM, in the extra-early Desmayo (D), the late Antoñeta (A), the extra-late Penta (P) and the ultra-late Tardona (T).

| **m/z** | **Molecular formula** | **Tentative identification** | **Adduct** | **Ionization mode** | **Data base** | **ΔPPM** | **Cultivar** |
| --- | --- | --- | --- | --- | --- | --- | --- |
| 70.0653 | C4H7N | 1-Pyrroline | [M+H]+ | Positive | Metlin | 2 | T |
| 80.0344 | CH5NO3 | Ammonium bicarbonate | [M+H]+ | Positive | Metlin | 2 | D |
| 85.0284 | C4H4O2 | 2(5H)-furanone | [M+H]+ | Positive | Metlin | 0 | D |
| 87.0092 | C3H4O3 | Pyruvate | [M-H]- | Negative | Metlin | 4 | P |
| 88.1122 | C5H13N | 2-Methylbutylamine | [M+H]+ | Positive | Metlin | 1 | D, P |
| 96.9613 | Unknown |  |  | Negative |  |  | A |
| 111.0444 | C6H8O3 | Methyl-4-oxo-2-pentenoate | [M+H-H2O]+ | Positive | Metlin | 1 | D, P |
| 116.0711 | C5H9NO2 | L-proline | [M+H]+ | Positive | HMDB | 4 | T |
| 123.0463 | C7H8O2 | 4-Methylcatechol | [M-H]- | Negative | Metlin | 9 | T |
| 127.0394 | C6H8O4 | 2,4-Dihydroxy-2,5-dimethyl-3(2H)-furanone | [M+H-H2O]+ | Positive | Metlin | 0 | D |
| 137.1330 | C10H18O | (±)-Borneol, (+)-isoborneol, 2-decenal | [M+H-H2O]+ | Positive | Metlin | 0 | P |
| 144.1021 | C7H15NO3 | L-carnitine | [M+H-H2O]+ | Positive | Metlin | 2 | P, T |
| 144.1387 | C8H17NO | Unknown | [M+H-H2O]+ | Positive | LipidMaps | 2 | A, P, T |
| 147.0440 | C9H8O3 | p-Coumaric acid | [M+H-H2O]+ | Positive | Metlin | 4 | P |
| 148.0608 | C5H9NO4 | D-glutamic acid | [M+H]+ | Positive | Metlin | 2 | T |
| 149.0236 | C8H6O4 | 3-Formylsalicylic acid | [M+H-H2O]+ | Positive | Metlin | 1 | P |
| 149.0597 | C9H8O2 | Cinnamic acid | [M+H+] | Positive | HMDB | 0 | P |
| 150.1127 | Unknown |  |  | Negative |  |  | D, A |
| 166.0870 | C9H11NO2 | Phenylalanine | [M+H+] | Positive | HMDB | 4 | P |
| 175.0236 | C6H6O6 | Dehydroascorbic acid | [M+H]+ | Positive | HMDB | 1 | A, T |
| 177.0411 | C6H8O6 | Ascobic acid | [M+H]+ | Positive | HMDB | 10 | D,A,P,T |
| 177.0550 | C10H8O3 | 4-Methylumbelliferone | [M+H]+ | Positive | Metlin | 2 | P |
| 181.0468 | C7H10O4 | Citraconic acid dimethyl ester | [M+Na]+ | Positive | Metlin | 1 | P |
| 181.0684 | C6H12O6 | D-glucose | [M+H]+ | Positive | HMDB | 13 | D |
| 181.0495 | C9H8O4 | Caffeic acid | [M+H+] | Positive | HMDB | 0 | P |
| 182.0819 | C9H11NO3 | Tyrosine | [M+H+] | Positive | HMDB | 4 | P |
| 183.0863 | C6H14O6 | D-sorbitol | [M+H]+ | Positive | Metlin | 0 | D |
| 183.0868 | C10H12N2 | Tryptamine | [M+Na]+ | Positive | HMDB | 13 | D |
| 188.1282 | C9H17NO3 | KAPA | [M+H]+ | Positive | Metlin | 0 | A |
| 191.0220 | C6H8O7 | Citric acid | [M-H]- | Negative | Metlin | 11 | T |
| 195.0657 | C10H10O4 | Ferulic acid | [M+H+] | Positive | HMDB | 3 | P |
| 205.0975 | C11H12N2O2 | L-tryptophan | [M+H]+ | Positive | HMDB | 2 | D |
| 207.0684 | C11H12O4 | Dimethylcaffeic acid | [M-H]- | Negative | Metlin | 10 | D |
| 211.1311 | C10H20O3 | Hydroxy-decanoic acid | [M+Na]+ | Positive | Metlin | 3 | P |
| 251.1647 | C15H22O3 | Abscisic alcohol | [M+H]+ | Positive | HMDB | 2 | P |
| 258.1093 | C10H17N3O6 | Ala-Glu-Gly | [M+H-H2O]+ | Positive | Metlin | 1 | A |
| 261.0375 | C6H13O9P | Beta-D-fructose 2-phosphate | [M+H]+ | Positive | HMDB | 2 | D |
| 263.0556 | C6H15O9P | Sorbitol-6-phosphate | [M+H]+ | Positive | HMDB | 11 | D |
| 265.1285 | C11H20O7 | (Z)-2-methyl-2-butene-1,4-diol 4-O-beta-D-Glucopyranoside | [M+H]+ | Positive | Metlin | 1 | T |
| 265.1438 | C15H20O4 | Abscisic acid | [M+H]+ | Positive | Metlin | 1 | P |
| 277.2148 | C16H30O2 | cis-7-Hexadecenoic acid | [M+Na]+ | Positive | Metlin | 3 | P |
| 282.1536 | Unknown |  |  | Negative |  |  | T |
| 287.0584 | C15H12O6 | Dihydrokaempferol | [M-H]- | Negative | HMDB | 8 | T |
| 287.2279 | C15H30O2 | Fatty acid | [M+FA-H] | Negative | LipidMaps | 11 | P |
| 291.1206 | C15H18O3 | Unknown | [M+FA-H] | Negative | Metlin | 10 | T |
| 293.1078 | C13H18O6 | Prunasin anitrile | [M+Na]+ | Positive | Standard |  | D,A,P,T |
| 293.1801 | C14H30O4S | Myrsinone | [M-H]- | Negative | Metlin | 14 | P |
| 295.1107 | C16H14N2O |  | [M+FA-H] | Negative | HMDB | 6 | T |
| 301.2041 | C15H28O3 | xi-7-Hydroxyhexadecanedioic acid | [M-H]- | Negative | HMDB | 6 | D |
| 308.0866 | C10H17N3O6S | Glutathione | [M+H]+ | Positive | HMDB | 15 | D,A,P,T |
| 315.2611 | C18H36O4 | Fatty acid | [M+FA-H] | Negative | LipidMaps | 13 | D, P |
| 318.0929 | C14H17NO6 | Prunasin | [M+Na]+ | Positive | Standard |  | D,A,P,T |
| 337.0980 | C14H18O8 | Prunasin acid | [M+Na]+ | Positive | Standard |  | D, A, P |
| 341.0066 | C6H14O12P2 | D-fructose 2,6-bisphosphate | [M+H]+ | Positive | HMDB | 10 | D |
| 361.1954 | C17H22N4O5 | Trp-Ala-Ser | [M-H]- | Negative | Predicted by DataAnalyst | 13 | D,A,P,T |
| 369.0943 | C19H16O5 |  | [M+FA-H]- | Negative | Metlin | 9 | D |
| 387.2113 | C23H32O5 | Fatty acid | [M-H]- | Negative | LipidMaps | 12 | D, A |
| 413.1566 | C22H24O5 |  | [M+FA-H]- | Negative | Metlin | 9 | A |
| 415.1354 | C21H22O6 |  | [M+FA-H]- | Negative | Metlin | 10 | A |
| 418.0755 | Unknown |  |  | Negative |  |  | A |
| 447.1282 | C22H22O10 | Glycitin / Hispidulin 7-rhamnoside | [M+H]+ | Positive | Metlin | 0 | P |
| 447.1667 | C22H26O7 |  | [M+FA-H] | Negative | Metlin | 7 | P |
| 461.2510 | C25H36O5 |  | [M+FA-H]- | Negative | Metlin | 7 | D |
| 477.1172 | C22H26O7 |  | [M+FA-H] | Negative | HMDB | 12 | A |
| 479.1163 | C22H22O12 | Petunidin 3-glucoside/galactoside | [M+H]+ | Positive | Metlin | 4 | D, P |
| 479.1224 | C21H22O10 | 4,5-Dihydroxy-2-(hydroxymethyl)-10-oxo-9,10-dihydro-9-anthracenyl hexopyranoside | [M+FA-H] | Negative | HMDB | 6 | A |
| 493.1354 | C23H25O12 | Malvidin 3-glucoside/galactoside | [M+H]+ | Positive | HMDB | 2 | D, P |
| 503.2626 | C29H36N4O4 | Ceanothine B | [M-H]- | Negative | HMDB | 8 | P, T |
| 503.3349 | C30H46O6 | Medicagenic acid | [M+H]+ | Positive | Metlin | 3 | P |
| 507.1474 | C21H28N6O6S2 | His-Tyr-Cys-Cys | [M+H-H2O]+ | Positive | Metlin | 2 | T |
| 517.1870 | Unknown |  |  | Negative |  |  | A |
| 517.3592 | C30H48O4 | Corosolic acid | [M+FA-H]- | Negative | Metlin | 2 | D |
| 519.1842 | C23H32N6O5S2 | Amino acidic compound | [M+H-H2O]+ | Positive | Metlin | 1 | P |
| 543.1823 | C22H28N6O9 | His-Asp-Tyr-Ser | [M+Na]+ | Positive | Metlin | 2 | D |
| 551.0975 | C24H22O15 | Quercetin 3-(6''-malonylglucoside) | [M+H]+ | Positive | Metlin | 10 | D |
| 551.1741 | C33H28O9 | Asticolorin C | [M+H-H2O]+ | Positive | Metlin | 6 | D |
| 553.2207 | C27H34N2O9 | Strictosidine | [M+Na]+ | Positive | Metlin | 9 | D |
| 563.1238 | C29H24O12 | Theaflavin | [M-H]- | Negative | Metlin | 7 | D |
| 563.1901 | C30H30O8 | Gossypol | [M+FA-H]- | Negative | Metlin | 3 | D |
| 567.3464 | Unknown |  |  | Negative |  |  | D |
| 575.1727 | C23H32N6O6S2 | Four amino acid peptide | [M+Na]+ | Positive | Metlin | 1 | D |
| 584.2724 | C29H39N5O9 | Amino acidic compound | [M+H-H2O]+ | Positive | Metlin | 0 | D, P |
| 601.4251 | C40H56O4 | Violaxanthin | [M+H]+ | Positive | HMDB | 0 | P |
| 623.1572 | C28H32O16 | Flavonoid | [M-H]- | Negative | LipidMaps | 7 | P |
| 707.2012 | C37H73O10P/ C44H84O6 | PG/TG/PE/PA | [M-H]- | Negative | LipidMaps | 20 | D, T |
| 759.4219 | C35H70O13P2/ C41H63O8P | PGP/PA | [M-H]- | Negative | LipidMaps | 16 | P |
| 889.2552 | C56H42O12 | Viniferol A | [M+H-H2O]+ | Positive | Metlin | 10 | P |

**Supplementary table S4.** Statistical values of all our metabolites after performing the volcano plot analysis. Key: orange cells correspond to Desmayo Largueta, purple cells correspond to Antoñeta, blue cells correspond to Penta and green cells correspond to Tardona.

| **Metabolite** | **t.stat** | **p.value** | **-LOG10(p)** | **FDR** | **Fold Change** | **log2(FC)** |
| --- | --- | --- | --- | --- | --- | --- |
| 1-Pyrroline | -1.2764 | 0.2490 | 0.6038 | 0.3652 | 4.2374 | 2.0832 |
| 2,4-Dihydroxy-2,5-dimethyl-32H-furanone | -2.5751 | 0.0497 | 1.3034 | 0.0900 | 5.3068 | 2.4078 |
| 25H-Furanone | -4.3412 | 0.0074 | 2.1296 | 0.0299 | 17.6980 | 4.1455 |
| 2-Methylbutylamine | -18.7130 | 8.02E-06 | 5.0956 | 0.0002 | 735.7400 | 9.5230 |
|  | -8.8163 | 0.0009 | 3.0394 | 0.0051 | 479.2700 | 8.9047 |
| 3-Formylsalicylic acid | 1.3962 | 0.2352 | 0.6286 | 0.3163 | 0.5844 | -0.7749 |
| 4,5-Dihydroxy-2-hydroxymethyl-10-oxo-9,10-dihydro-9-anthracenyl hexopyranoside | 5.9048 | 0.0041 | 2.3855 | 0.0156 | 0.3647 | -1.4553 |
| 4-Methylcatechol | 1.3846 | 0.2384 | 0.6227 | 0.3020 | 0.7559 | -0.4038 |
|  | 0.1760 | 0.8661 | 0.0624 | 0.9012 | 1.3134 | 0.3933 |
| 4-Methylumbelliferone | 1.7432 | 0.1563 | 0.8062 | 0.2257 | 0.4777 | -1.0658 |
| Aas1 | 2.0717 | 0.0930 | 1.0314 | 0.1309 | 0.2097 | -2.2533 |
| Aas2 | -6.1880 | 0.0016 | 2.7939 | 0.0148 | 376.4800 | 8.5564 |
| Abscisic acid | 0.9361 | 0.4022 | 0.3955 | 0.4902 | 0.2312 | -2.1125 |
| Abscisic alcohol | -0.3647 | 0.7338 | 0.1344 | 0.7735 | 1.8187 | 0.8629 |
| Ala Glu Gly | -5.1083 | 0.0069 | 2.1585 | 0.0193 | 2.1772 | 1.1225 |
| Aminoacid compound | 5.4520 | 0.0055 | 2.2597 | 0.0179 | 0.3148 | -1.6676 |
| Aminoacid compound, it begins with hys | -49.0880 | 1.03E-06 | 5.9869 | 4.02E-05 | 8294.8000 | 13.0180 |
| Ammonium bicarbonate | 0.9562 | 0.3829 | 0.4169 | 0.4042 | 0.5668 | -0.8192 |
| Ascorbic Acid | -1.8667 | 0.1209 | 0.9175 | 0.1532 | 2.6066 | 1.3822 |
|  | -4.8475 | 0.0084 | 2.0781 | 0.0198 | 2.9080 | 1.5400 |
|  | -6.7903 | 0.0025 | 2.6097 | 0.0087 | 62.5980 | 5.9681 |
|  | -0.2388 | 0.8192 | 0.0866 | 0.9012 | 1.1224 | 0.1666 |
| Asticolorin C | 2.5004 | 0.0545 | 1.2639 | 0.0941 | 0.3108 | -1.6858 |
| Beta-D-Fructose 2-phosphate | -1.7191 | 0.1462 | 0.8349 | 0.1793 | 2.3474 | 1.2310 |
| Borneol, Isoborneol, 2-decenal | 3.0736 | 0.0372 | 1.4299 | 0.0665 | 0.3719 | -1.4270 |
| C15H18O3 | 4.7748 | 0.0031 | 2.5115 | 0.0169 | 0.2306 | -2.1165 |
| C16H14N2O | 2.6524 | 0.0379 | 1.4213 | 0.0834 | 0.2366 | -2.0794 |
| C19H16O5 | 1.1872 | 0.2885 | 0.5399 | 0.3132 | 0.5638 | -0.8268 |
| C20H28O3 | 4.2622 | 0.0080 | 2.0970 | 0.0299 | 0.3489 | -1.5192 |
|  | 5.0755 | 0.0071 | 2.1485 | 0.0193 | 0.3796 | -1.3974 |
|  | 8.5094 | 0.0010 | 2.9804 | 0.0051 | 0.1255 | -2.9941 |
|  | 8.6783 | 0.0001 | 3.8888 | 0.0028 | 0.1660 | -2.5908 |
| C21H22O6 | 1.7870 | 0.1485 | 0.8284 | 0.2351 | 0.4223 | -1.2436 |
| C22H24O5 | 1.4611 | 0.2178 | 0.6620 | 0.2956 | 0.4109 | -1.2831 |
| C22H26O7 | 1.2723 | 0.2722 | 0.5651 | 0.3232 | 0.7851 | -0.3491 |
|  | -1.6122 | 0.1822 | 0.7394 | 0.2538 | 4.4835 | 2.1646 |
| C25H36O5 | 3.8947 | 0.0115 | 1.9404 | 0.0335 | 0.2484 | -2.0092 |
| C8H17NO | -3.4761 | 0.0254 | 1.5945 | 0.0483 | 4.0613 | 2.0220 |
|  | -3.8360 | 0.0185 | 1.7323 | 0.0380 | 22.1410 | 4.4687 |
|  | -5.3218 | 0.0018 | 2.7466 | 0.0131 | 4.4373 | 2.1497 |
| Caffeic acid | -6.9513 | 0.0023 | 2.6478 | 0.0087 | 47.6990 | 5.5759 |
| Ceanothine B | 1.3620 | 0.2449 | 0.6111 | 0.3183 | 0.5793 | -0.7876 |
|  | 4.3487 | 0.0048 | 2.3161 | 0.0213 | 0.3563 | -1.4888 |
| Cinnamic acid | -4.1421 | 0.0144 | 1.8431 | 0.0311 | 11.4760 | 3.5205 |
| cis-7-Hexadecenoic Acid | 5.1291 | 0.0068 | 2.1648 | 0.0191 | 0.2664 | -1.9085 |
| Citraconic acid dimethyl ester | -6.9513 | 0.0023 | 2.6478 | 0.0087 | 47.6990 | 5.5759 |
| Citric acic | 3.4390 | 0.0138 | 1.8596 | 0.0410 | 0.2891 | -1.7904 |
| Corosolic acid | 4.1793 | 0.0087 | 2.0625 | 0.0299 | 0.3316 | -1.5923 |
| Dehydroascorbate | -1.0307 | 0.3609 | 0.4426 | 0.3810 | 2.9212 | 1.5466 |
|  | 1.8682 | 0.1110 | 0.9548 | 0.1878 | 0.5347 | -0.9033 |
| D-Fructose 2,6-bisphosphate | -1.3154 | 0.2455 | 0.6100 | 0.2744 | 2.0625 | 1.0444 |
| D-Glucose | 2.6656 | 0.0446 | 1.3508 | 0.0900 | 0.3952 | -1.3393 |
| D-Glutamic acid | 1.9480 | 0.0993 | 1.0029 | 0.1878 | 0.6622 | -0.5947 |
| Dihydrokaempferol | -0.2134 | 0.8381 | 0.0767 | 0.9012 | 1.3527 | 0.4359 |
| Dimetylcaffeic acid | -5.0877 | 0.0038 | 2.4191 | 0.0211 | 5.8749 | 2.5546 |
| FA 1 | 7.6408 | 0.0016 | 2.8024 | 0.0100 | 0.4157 | -1.2664 |
|  | 0.9060 | 0.4162 | 0.3807 | 0.4918 | 0.5841 | -0.7758 |
| FA 2 | 3.8996 | 0.0114 | 1.9426 | 0.0335 | 0.2155 | -2.2146 |
| FA 3 | 3.2661 | 0.0223 | 1.6518 | 0.0529 | 0.2779 | -1.8472 |
| Fatty acid | -1.2165 | 0.2907 | 0.5366 | 0.3657 | 1.4454 | 0.5314 |
| Ferulic acid | -5.3302 | 0.0060 | 2.2244 | 0.0179 | 22.7570 | 4.5083 |
| Flavonoid | -0.1122 | 0.9161 | 0.0381 | 0.9161 | 1.2260 | 0.2940 |
| Glutathione | -1.6177 | 0.1667 | 0.7782 | 0.1919 | 2.1722 | 1.1192 |
|  | -0.2259 | 0.8324 | 0.0797 | 0.8324 | 0.8948 | -0.1604 |
|  | -0.5655 | 0.6019 | 0.2205 | 0.6707 | 2.1824 | 1.1259 |
|  | 1.8721 | 0.1104 | 0.9572 | 0.1878 | 0.3323 | -1.5896 |
| Glycitin o Hispidulin 7-rhamnoside | 3.0635 | 0.0375 | 1.4256 | 0.0665 | 0.3803 | -1.3950 |
| Gossypol | 7.2736 | 0.0008 | 3.1146 | 0.0097 | 0.4045 | -1.3056 |
| His Asp Tyr Ser | 2.3735 | 0.0637 | 1.1960 | 0.1008 | 0.2575 | -1.9571 |
| His Tyr Cys Cys | 3.3766 | 0.0149 | 1.8262 | 0.0410 | 0.4079 | -1.2936 |
| hydroxy-decanoic acid | 4.4833 | 0.0110 | 1.9601 | 0.0285 | 0.4589 | -1.1237 |
| KAPA | -9.3919 | 0.0007 | 3.1450 | 0.0068 | 5.5833 | 2.4811 |
| L-Carnitine | 1.8179 | 0.1432 | 0.8440 | 0.2148 | 0.5481 | -0.8676 |
|  | 5.9338 | 0.0010 | 2.9904 | 0.0112 | 0.1513 | -2.7248 |
| L-Proline | 0.1332 | 0.8984 | 0.0465 | 0.9012 | 1.0965 | 0.1329 |
| L-Tryptophan | -4.8824 | 0.0045 | 2.3426 | 0.0216 | 3.8562 | 1.9472 |
| Malvidin 3-glucoside | 0.6198 | 0.5625 | 0.2499 | 0.5625 | 0.7003 | -0.5140 |
|  | 0.1287 | 0.9038 | 0.0439 | 0.9161 | 1.3888 | 0.4739 |
| Medicagenic acid | 1.8280 | 0.1416 | 0.8491 | 0.2148 | 0.6330 | -0.6598 |
| Methyl 4-oxo-2-pentenoate | 2.6068 | 0.0479 | 1.3201 | 0.0900 | 0.1542 | -2.6976 |
| Myrsinone | -0.4730 | 0.6609 | 0.1799 | 0.7159 | 1.8599 | 0.8952 |
| p-Coumaric acid | -23.6470 | 1.90E-05 | 4.7221 | 0.0003 | 279.7600 | 8.1281 |
| Petunidin 3-glucoside | -2.1191 | 0.0876 | 1.0575 | 0.1280 | 10.6300 | 3.4100 |
|  | -12.6960 | 0.0002 | 3.6543 | 0.0014 | 288.9700 | 8.1748 |
| PG/TG/PE/PA | 1.9750 | 0.1053 | 0.9778 | 0.1379 | 0.2781 | -1.8463 |
|  | -0.4314 | 0.6812 | 0.1667 | 0.8816 | 0.7743 | -0.3690 |
| PGP/PA | 4.1881 | 0.0138 | 1.8593 | 0.0311 | 0.0421 | -4.5713 |
| Phenylalanine | -4.2776 | 0.0129 | 1.8903 | 0.0311 | 3.3070 | 1.7255 |
| Prunasin | -5.0649 | 0.0039 | 2.4107 | 0.0211 | 8.5933 | 3.1032 |
|  | -6.5814 | 0.0028 | 2.5592 | 0.0131 | 90.0980 | 6.4934 |
|  | -18.5470 | 4.97E-05 | 4.3033 | 0.0005 | 90.1200 | 6.4938 |
|  | -1.1756 | 0.2843 | 0.5462 | 0.3909 | 11.0880 | 3.4709 |
| Prunasin acid | 2.0081 | 0.1009 | 0.9961 | 0.1369 | 0.4039 | -1.3080 |
|  | -14.1760 | 0.0001 | 3.8424 | 0.0011 | 4.4914 | 2.1672 |
|  | 1.5221 | 0.1788 | 0.7476 | 0.2810 | 0.5520 | -0.8573 |
| Prunasin anitrile | 335.5200 | 4.46E-12 | 11.3500 | 1.70E-10 | 0.3712 | -1.4297 |
|  | 1.1600 | 0.3106 | 0.5079 | 0.3471 | 0.7605 | -0.3949 |
|  | -21.8740 | 2.58E-05 | 4.5876 | 0.0003 | 1.2782 | 0.3542 |
|  | -0.1294 | 0.9012 | 0.0452 | 0.9012 | 1.0706 | 0.0984 |
| Pyruvate | -0.6918 | 0.5271 | 0.2781 | 0.6046 | 1.8268 | 0.8694 |
| Quercetin 3-6''-malonylgalactoside | -2.8690 | 0.0350 | 1.4555 | 0.0783 | 3.6681 | 1.8750 |
| Sorbitol | 3.5799 | 0.0159 | 1.7993 | 0.0426 | 0.0551 | -4.1827 |
| Sorbitol-6-phosphate | -2.3900 | 0.0624 | 1.2049 | 0.1008 | 7.1861 | 2.8452 |
| Strictosidine | 0.7624 | 0.4802 | 0.3186 | 0.4932 | 0.8388 | -0.2536 |
| Theaflavin | -1.6273 | 0.1646 | 0.7836 | 0.1919 | 1.6334 | 0.7079 |
| Tryptamine | 3.5252 | 0.0168 | 1.7740 | 0.0426 | 0.0567 | -4.1395 |
| Tyrosine | -3.5122 | 0.0246 | 1.6087 | 0.0480 | 8.2583 | 3.0458 |
| Unknown 3 | 1.5029 | 0.2073 | 0.6834 | 0.2956 | 0.5893 | -0.7630 |
| Unknown 4 | 2.2836 | 0.0845 | 1.0734 | 0.1459 | 0.5228 | -0.9356 |
| Viniferol A | 2.1292 | 0.1003 | 0.9987 | 0.1630 | 0.5058 | -0.9833 |
| Violaxanthin | 2.4315 | 0.0719 | 1.1435 | 0.1219 | 0.5977 | -0.7425 |
| xi-7-Hydroxyhexadecanedioic acid | 5.9320 | 0.0019 | 2.7116 | 0.0148 | 0.1804 | -2.4706 |
| Z-2-Methyl-2-butene-1,4-diol 4-O-beta-D-Glucopyranoside | 4.1041 | 0.0063 | 2.1987 | 0.0232 | 0.2049 | -2.2871 |
| Unknown 1 | 2.3395 | 0.0664 | 1.1777 | 0.1010 | 0.1463 | -2.7727 |
|  | 15.1550 | 0.0001 | 3.9566 | 0.0021 | 0.0412 | -4.6006 |
|  | 3.2373 | 0.0177 | 1.7509 | 0.0434 | 0.2130 | -2.2311 |
| Unknown 2 | 2.5945 | 0.0486 | 1.3136 | 0.0900 | 0.3358 | -1.5744 |
|  | 4.6419 | 0.0097 | 2.0123 | 0.0205 | 0.2480 | -2.0117 |


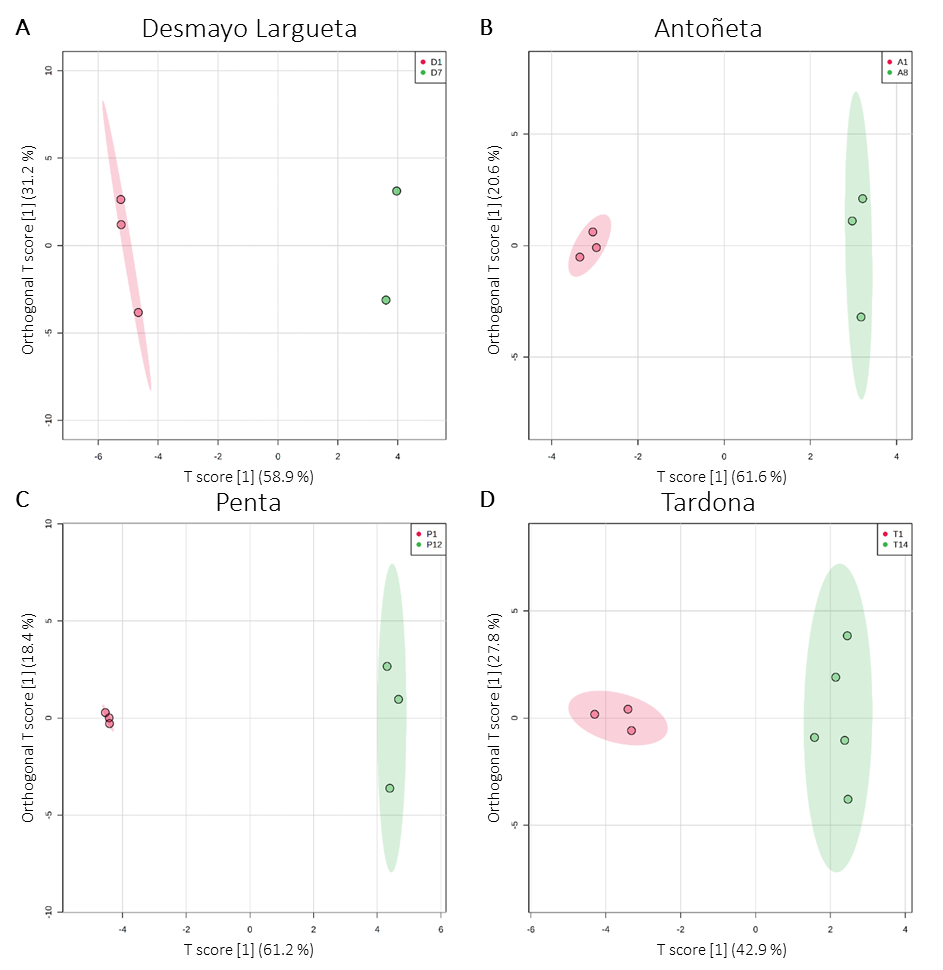


**Supplementary Figure S1.** OPLS-DA between endodormant and ecodormant flower buds showing the 95% confidence region in the four cultivars studied: **A** the extra-early Desmayo Largueta, **B** the late Antoñeta, **C** the extra-late Penta and **D** the ultra-late Tardona. Key: Red circles correspond to endodormant flower buds and green circles correspond to ecodormant flower buds. The R^2^X, R^2^Y and Q^2^ of both T score and orthogonal T score components are shown in Supplementary Figure S3.


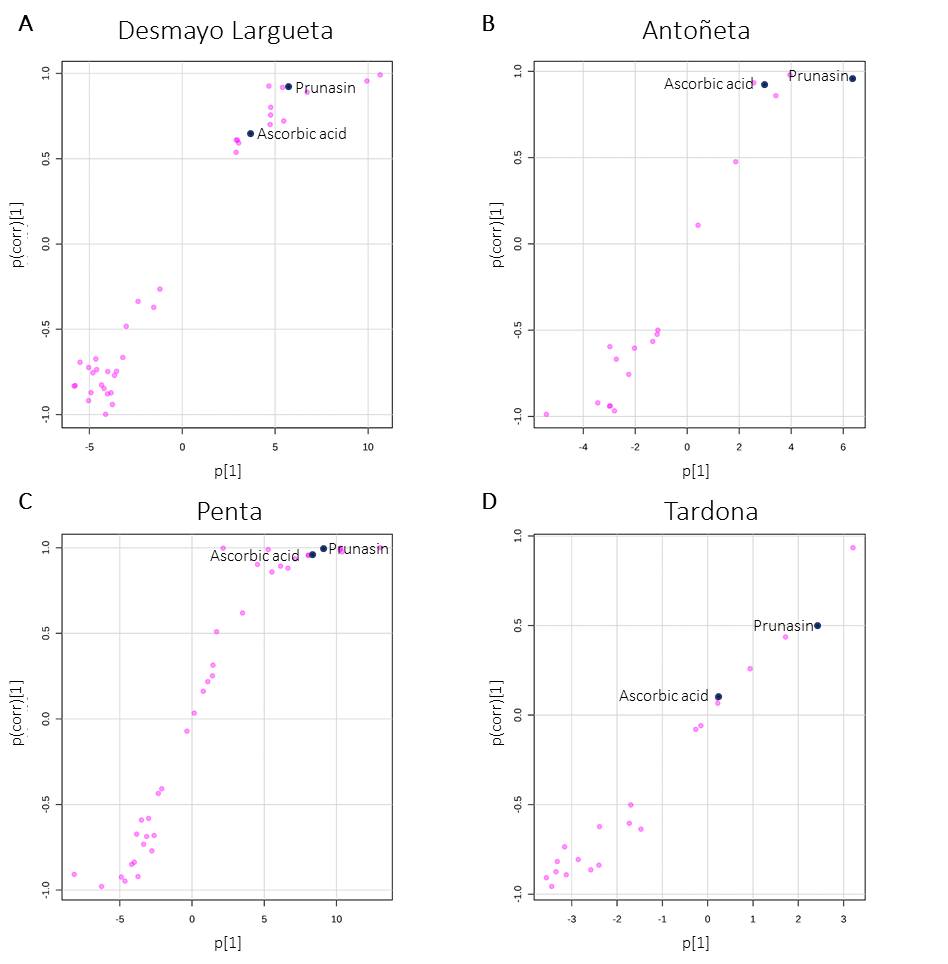


**Supplementary Figure S2.** Importance feature plot of the OPLS-DA in the four cultivars studied: the extra-early Desmayo Largueta (A), the late Antoñeta (B), the extra-late Penta (C) and the ultra-late Tardona cultivar (D). Prunasin and ascorbic acid are localized in the top right of the A, B and C plots, resembling their importance in the model construction. In the ultra-late Tardona cultivar (D) only, ascorbic acid is present in the middle of the plot, but prunasin is found in the top right. Key: Dark blue points correspond to the two metabolites that varied significantly in all our cultivars (prunasin and ascorbic acid).


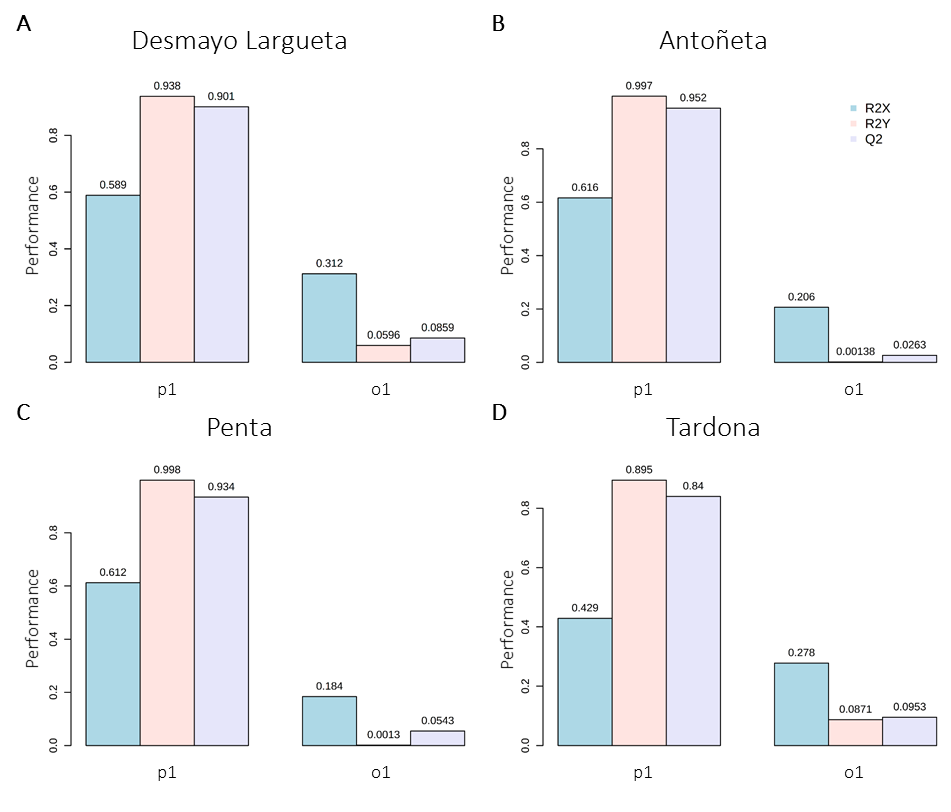


**Supplementary Figure S3.** OPLS-DA overview in the four cultivars studied: the extra-early Desmayo Largueta (A), the late Antoñeta (B), the extra-late Penta (C) and the ultra-late Tardona cultivar (D). For every cultivar, the R^2^Y and the prediction capability (Q^2^) from the T score values (p1) are higher than in the orthogonal one (o1), meaning that p1 has a greater impact and reliability in the model than o1.


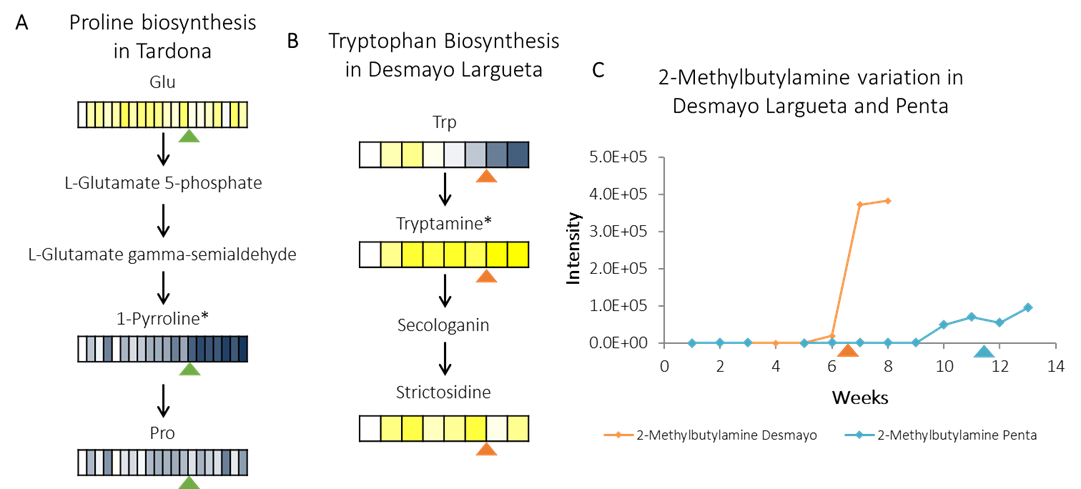


**Supplementary Figure S4. A**. Amino acid biosynthesis of Pro. A heatmap showing the variation of Glu, 1-pyrroline and Pro across the twenty weeks from endo- to ecodormancy in the ultra-late Tardona cultivar. **B**. Trp metabolism. A heatmap showing the variation of Trp, the intermediate tryptamine and strictosidine across the eight weeks in the extra-early Desmayo Largueta cultivar. Values represent the means of FC from three biological replicates. Asterisks show the metabolites whose variation meets the requirements for being considered significant. **C**. Methylbutylamine variation in the extra-early Desmayo Largueta (orange) cultivar and the extra-late Penta (blue) cultivar. Triangles marks the dormancy release date.


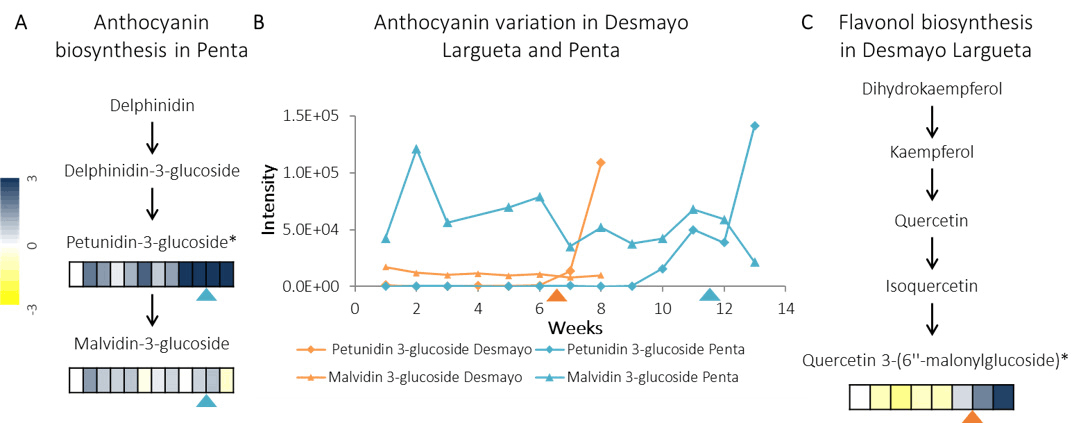


**Supplementary Figure S5. A.** A heatmap showing the variation of two anthocyanins petunidin-3-glucoside and malvidin-3-glucoside across the twelve weeks from endodormancy to ecodormancy in the extra-late cultivar Penta Values represent the means of FC from three biological replicates. **B.** Petunidin-3-glucoside (diamond) and malvidin 3-glucoside (triangle) content variation from endo- to ecodormancy in the extra-early Desmayo Largueta (orange) and in the extra-late Penta (blue) cultivar. Petunindin 3-glucoside presented an FC over 3, while malvidin-3-glucoside did not behave in the same way. Triangles marks the dormancy release date. **C.** Flavonol biosynthesis. A heatmap showing the variation of quercetin 3-(6”-malonylglucoside) across the eight weeks assayed from endo- to ecodormancy in the extra-early Desmayo Largueta cultivar. Values represent the means of FC from three biological replicates in the extra-early Desmayo Largueta and the ultra-late Tardona. Asterisks show the metabolites whose variation meets the requirements for being considered significant.


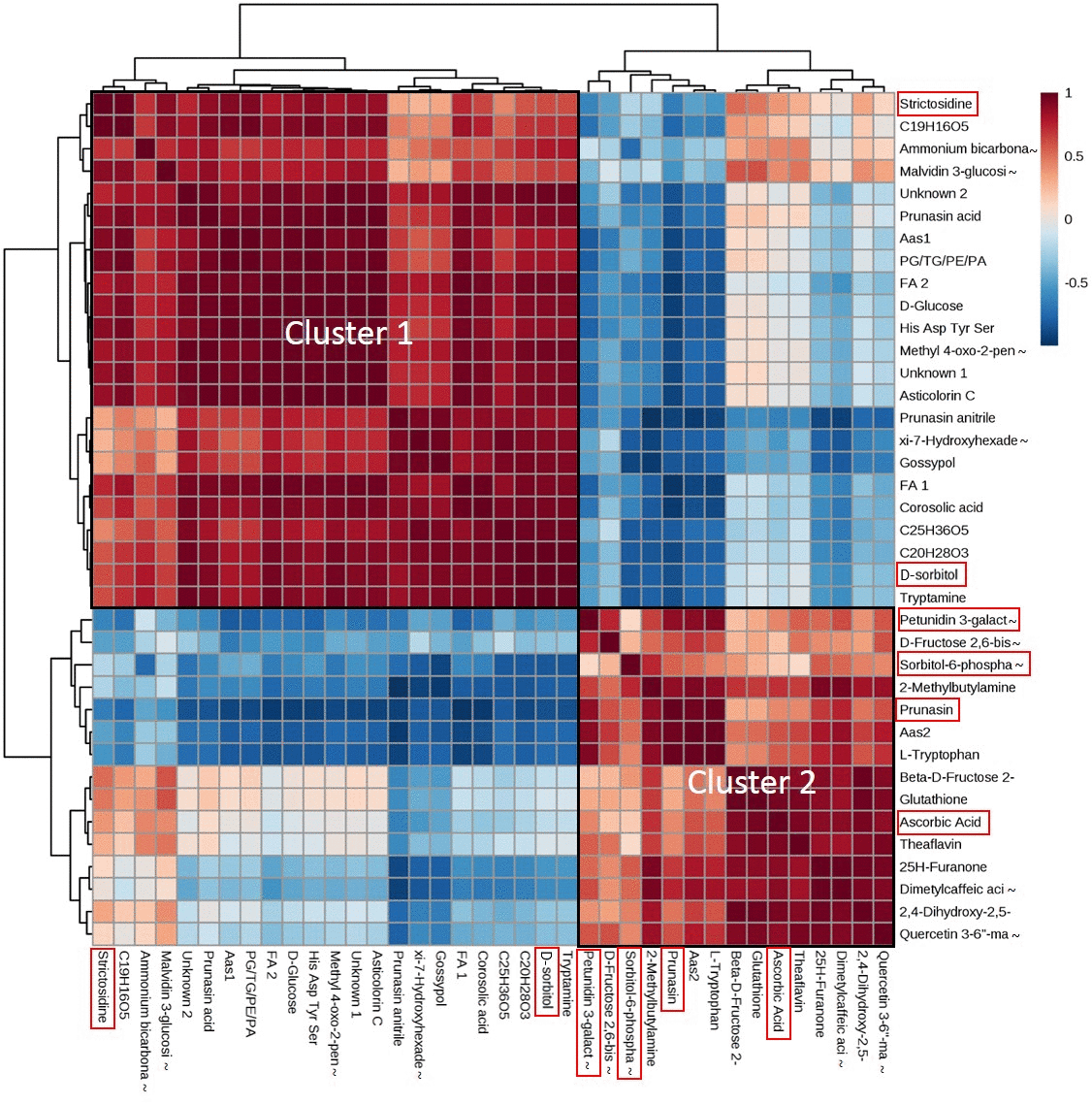


**Supplementary Figure S6.** A correlation heatmap of the extra-early Desmayo Largueta cultivar, showing two different clusters of metabolites. In general, metabolites of the same pathway grouped together in the same cluster. The complete names of metabolites with ~ can be found in Supplementary Table S3.


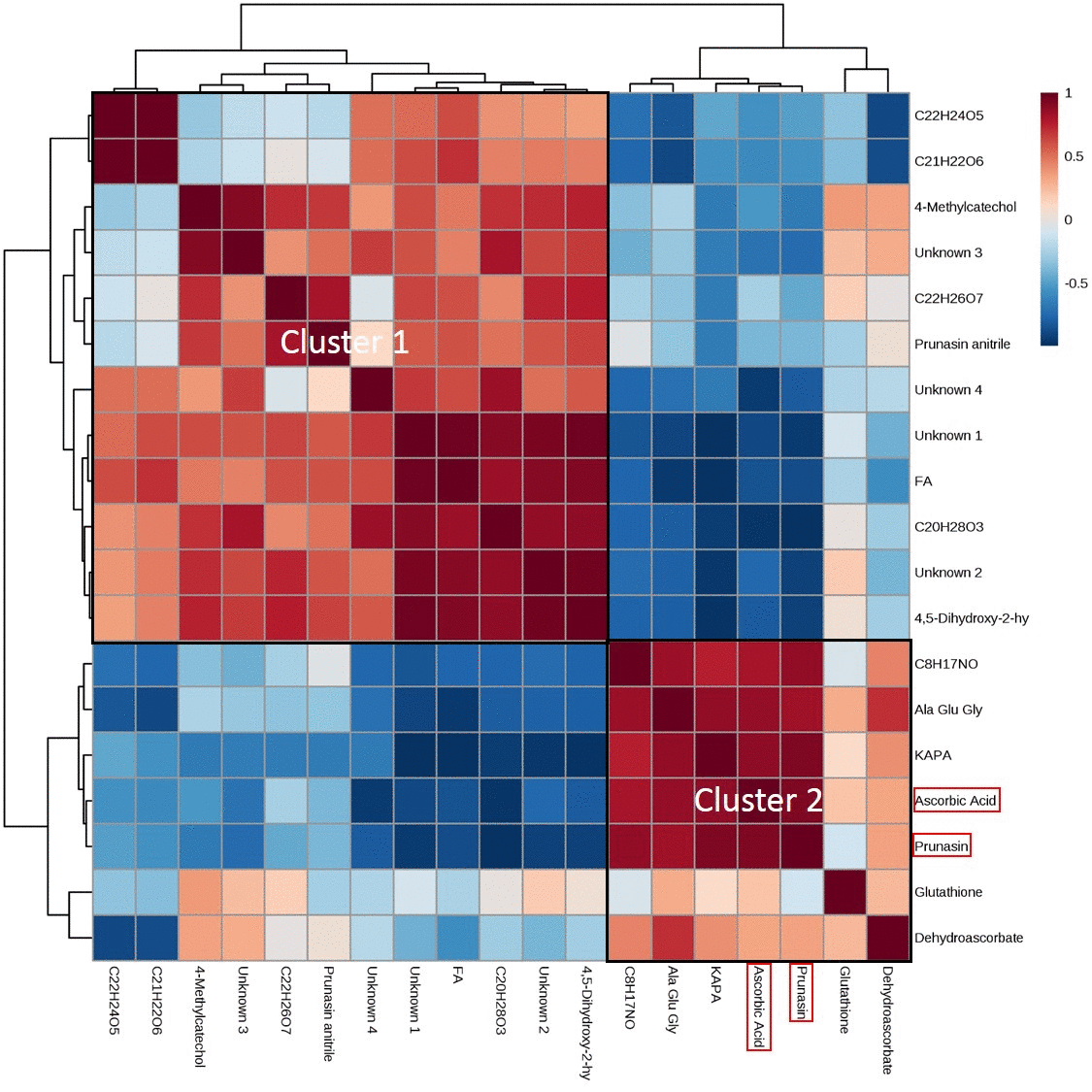


**Supplementary Figure S7.** A correlation heatmap of the late Antoñeta cultivar, where two clusters are also found. Metabolites of the same pathway grouped together in the same cluster. The complete name of the metabolites with ~ can be found in Supplementary Table S3.


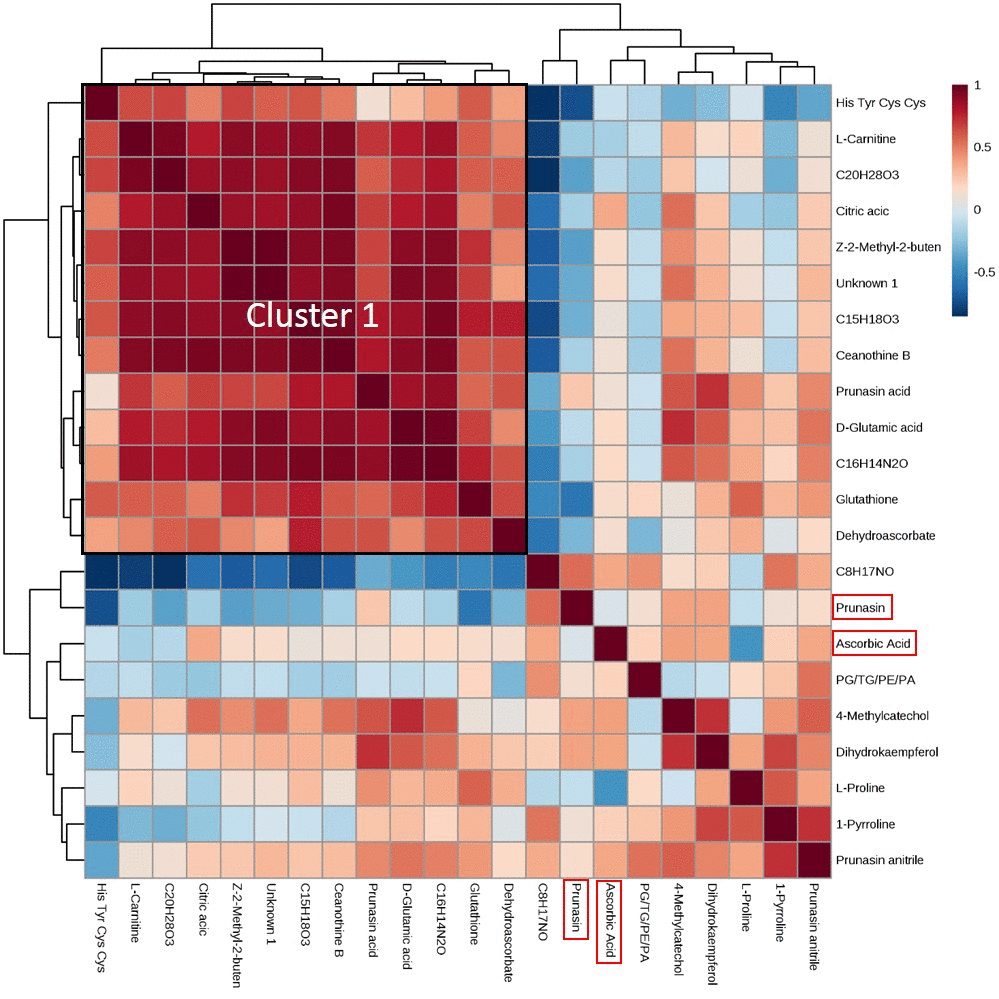


**Supplementary Figure S8.** A correlation heatmap of the ultra-late Tardona cultivar, showing only one cluster. The complete name of the metabolites with ~ can be found in Supplementary Table S3.
